# Immunomodulation Eliminates Inflammation in the Hippocampus in Experimental Autoimmune Encephalomyelitis, but Does Not Ameliorate Anxiety-Like Behavior

**DOI:** 10.3389/fimmu.2021.639650

**Published:** 2021-06-10

**Authors:** Pece Kocovski, Nuzhat Tabassum-Sheikh, Stephanie Marinis, Phuc T. Dang, Matthew W. Hale, Jacqueline M. Orian

**Affiliations:** ^1^ Department of Psychology and Counselling, School of Psychology and Public Health, La Trobe University, Melbourne, VIC, Australia; ^2^ Department of Biochemistry and Genetics, La Trobe Institute for Molecular Science (LIMS), La Trobe University, Melbourne, VIC, Australia

**Keywords:** EAE (experimental autoimmune encephalomyelitis), hippocampus, immunomodulation, multiple sclerosis, anxiety and major depression, S1PR, EPM (elevated plus maze), FTY720 (fingolimod)

## Abstract

Multiple sclerosis (MS) is an autoimmune disease targeting the central nervous system, characterized by an unpredictable disease course and a wide range of symptoms. Emotional and cognitive deficits are now recognized as primary disease manifestations and not simply the consequence of living with a chronic condition, raising questions regarding the efficacy of current therapeutics for these specific symptoms. Mechanisms underlying psychiatric sequelae in MS are believed to be similar to those underlying pathogenesis, that is mediated by cytokines and other inflammatory mediators. To gain insight into the pathogenesis of MS depression, we performed behavioral assays in the murine experimental autoimmune encephalomyelitis (EAE) MS model, in the presence or absence of immunomodulation using the drug FTY720, an analogue of the lipid signaling molecule sphingosine-1-phosphate (S1P). Specifically, mice were challenged with the elevated plus maze (EPM) test, a validated experimental paradigm for rodent-specific anxiety-like behavior. FTY720 treatment failed to ameliorate anxiety-like symptoms, irrespective of dosage. On the other hand, it was effective in reducing inflammatory infiltration, microglial reactivity and levels of pro-inflammatory molecules in the hippocampus, confirming the anti-inflammatory capacity of treatment. To explore the absence of FTY720 effect on behavior, we confirmed expression of S1P receptors (S1PR) S1PR1, S1PR3 and S1PR5 in the hippocampus and mapped the dynamics of these receptors in response to drug treatment alone, or in combination with EAE induction. We identified a complex pattern of responses, differing between (1) receptors, (2) dosage and (3) hippocampal sub-field. FTY720 treatment in the absence of EAE resulted in overall downregulation of S1PR1 and S1PR3, while S1PR5 exhibited a dose-dependent upregulation. EAE induction alone resulted in overall downregulation of all three receptors. On the other hand, combined FTY720 and EAE showed generally no effect on S1PR1 and S1PR3 expression except for the fimbrium region, but strong upregulation of S1PR5 over the range of doses examined. These data illustrate a hitherto undescribed complexity of S1PR response to FTY720 in the hippocampus, independent of drug effect on effector immune cells, but simultaneously emphasize the need to explore novel treatment strategies to specifically address mood disorders in MS.

## Introduction

Multiple sclerosis is an autoimmune disease of the central nervous system (CNS) of unknown cause, manifesting most commonly in young adults (1). It is characterized by a very complex pathophysiology, which underscores the unpredictable course of the disease and implicates a wide range of CNS regions, resulting in an array of symptoms (2). Among these, anxiety and depression are significantly elevated relative the general population (3, 4), with around 30% of affected individuals being diagnosed with anxiety (5) and 50% reporting major depression at any point in their lifetime (6) associated with high risks of suicide, or suicidal thoughts (7). Until relatively recently, these symptoms were interpreted as a consequence of living with a chronic condition; however, over the last two decades evidence that depression can precede onset of neurological signs has accumulated, leading to growing exploration of potential primary mechanisms underlying depressive symptoms in MS (8).

The hippocampus is one of major brain regions associated with emotional behavior, cognition and memory. It is a compound structure located within the medial temporal lobes and showing broad, but not absolute specialization along the longitudinal axis (9). In MS, post-mortem histological investigations have identified this region as a major target for the disease, characterized by significant demyelination, volume loss and reduced synaptic density (10, 11). Imaging studies have revealed variability in the extent to which different hippocampal subfields exhibit damage or repair (12–16). In previous studies we investigated the accumulation of pathology in the mouse hippocampus using experimental autoimmune encephalomyelitis (EAE), a neuroinflammatory disease induced in rodents and non-human primates by vaccination with CNS proteins or peptides, which mimics facets of MS. We identified a complex pathological pattern (17) with differential levels of inflammation from dorsal to ventral plane and rostro to caudal directions. The fimbrium was the most severely affected and characterized by extensive CD3-positive cell infiltration, severe microglial reactivity and upregulation of α B-crystallin, a marker of CNS stress. On the other hand, other sub-fields including the CA1 and the dentate gyrus exhibited minor single CD3 cell entry, without evidence of lesions, but widespread microglial reactivity. Therefore, the pathology of the hippocampus in EAE recapitulates that of MS, by exhibiting severe white matter lesions, but microglial reactivity in the absence of major lymphocytic infiltration in grey matter.

The literature reports overall beneficial effects of MS immunomodulatory treatment, including FTY720 (18, 19) (Fingolimod, Gilenya^®^) on depression (20, 21). This therapeutic acts as a receptor modulator on four of the five members of the S1PR family (S1PR1, S1PR3, S1PR4 and S1PR5) in the form of its active metabolite FTY720-phosphate (FTY720-P). FTY720 has been studied most extensively for its effect on lymphocyte migration *via* S1PR1 and has been shown to prevent egress of these cells from lymphoid organs into efferent blood and lymph, thereby blocking migration of autoreactive effector T cells to sites of inflammation. Due to the wide S1PR distribution, FTY720 effects on other immune subsets and other organs including the CNS, have been demonstrated (22, 23). FTY720 efficacy on relapse rate, disability progression and magnetic resonance imaging parameters of lesion activity has been demonstrated in multiple clinical trials (24, 25). Several studies have also demonstrated the effect of FTY720 on EAE amelioration (26), while others have addressed the effects of this therapeutic on individual members of the S1PR family (27).

A number of studies suggest a role for proinflammatory mediators in mood disorders, therefore, to gain insight into the pathogenesis of anxiety and depression in MS, we performed behavioral analysis on EAE-induced mice, in the presence or absence of FTY720. This was achieved using the EPM test, which is a validated quantifiable test for anxiety-like behavior in mice (28) and meets the essential criteria of face validity (phenomenological similarity of the animal condition to the condition it is supposed to model), construct validity (a theoretical rationale underlying the model consistent with current knowledge of the human condition) and predictive validity (manipulations known to influence the human condition, such as drug effects, have an equivalent effect on the model) (28–31). This test has been used successfully in our hands (32, 33) and that of other investigators (34, 35) in evaluation of depressive-like behavioral syndrome due to MS. Additionally, this investigation was performed over a range of drug doses and the dynamics of S1PR in response to treatment were mapped.

## Materials and Methods

### EAE Induction

C57BL/6J mice were obtained from the Animal Resource Centre (Perth, Australia) and housed under standard conditions with food and water *ad libitum*. All procedures were approved by the institutional Animal Ethics Committee and performed strictly in accordance with regulations set by the National Health and Medical Research Council of Australia. Only female mice aged 12–16 weeks were used (32). EAE was performed as previously described (36). Briefly, mice received 200 µg of peptide 35-55 of myelin oligodendrocyte glycoprotein (MOG_35-55_), emulsified in complete Freund’s adjuvant (Sigma-Aldrich, St. Louis, MO) supplemented with 4 mg/ml of heat inactivated *Mycobacterium tuberculosis* (Becton Dickinson, Franklin Lakes, NJ) and administered subcutaneously. This was immediately followed by an intraperitoneal injection of 350 ng of *Bordetella pertussis* toxin (Sigma-Aldrich), which was repeated 48 hours later. Control groups included sham where MOG_33–55_ was substituted with phosphate buffered saline (PBS, 0.01 M phosphate, 15 mM NaCl, pH 7.4) and normal mice. Clinical progression was followed by daily weighing and visual assessment of ambulatory difficulties, scored as follows: 0 = no symptoms, 1= limp tail, 2 = hind limb weakness, 3 = hind limb paralysis, 4 = ascending paralysis, and 5 = moribund (36). The experimental design is summarized in **Supplementary Figure 1**.

### FTY720 Drug Delivery

FTY720 (also known as Fingolimod) was provided by Novartis Pharmaceuticals (Basel, Switzerland) and administered from day 6 of EAE *via* the drinking water, at a dosage of 0.7 mg/kg (FTY720-H) or 0.3 mg/kg (FTY720-L) body weight. Water bottles were weighed daily to calculate average daily water consumption. Experimental groups included EAE+FTY720-L, EAE+FTY720-H, sham+FTY720-L and sham+FTY720-H mice.

### EPM Test

The EPM consists of a central platform (5 x 5 cm) with four branching arms (30 x 5 cm each) at right angles to each other, where one pair of opposite arms is walled and the other open (37). The test was conducted at 9 days post induction (dpi) in a soundproof room under dim red lighting (40-41 lux) for 5 minutes, as previously described (32). Behavior was recorded using a HD webcam connected by a PC, by an investigator blinded as to mouse identity and treatment conditions.

### RNA Isolation, cDNA Synthesis and qPCR Analysis

Following transcardiac perfusion with ice-cold PBS, the whole brain was removed and the region containing the dorsal hippocampus (approximately –0.94 to -3.88 mm bregma) was sectioned using a brain matrix (Ted Pella Inc., Redding CA). The dorsal hippocampus was then collected using a biopsy punch, 1.5 mm in diameter. RNA was extracted *via* the Isolate II RNA Mini Kit RNA (BIO-52072, Bioline, MA) as recommended by the manufacturer and the quality of RNA preparations verified on a Microchip Electrophoresis System for DNA/RNA analysis, MCE-202 (MultiNa, Shimadzu Corporation, Japan). This was followed by cDNA synthesis using the Tetro cDNA Synthesis kit (BIO-65043, Bioline), as recommended by the manufacturer. Primer pairs for target genes (TNF-α, IFN-γ, S1PR1, S1PR3, and S1PR5) and reference genes (HSP90, GAPDH and βactin) are shown in **Table 1**.

**Table 1:** Target genes and their primer pairs.

Target gene	Forward primer sequence (5’-3’)	Reverse primer sequence (5’-3’)
IFNγ	TCATGGCTGTTTCTGGCTGT	CCCAGATACAACCCCGCAAT
TNFα	AAGCCTGTAGCCCACGTCGTA	GGCACCACTAGTTGGTTGTCTTTG
S1PR1	GCATTGTCAAGCTCCCAGAG	GAAGAAATGGAGGGTGGGGA
S1PR3	GTCTCCACAGGTCAAGCTCT	CGGGCTGAAATGTATCGGTG
S1PR5	CTCCAACAGTTTGCAGCGAT	TGGGAAGCGTCAGTCTGTAG
HSP90	GCTTTCCCGTCAAGATGCCT	CACCACTTCCTTGACCCTCC
GAPDH	GCTCATGACCACAGTCCATGC	GTTGGGATAGGGCCTCTCTTG
βactin	AGTGTGACGTTGACATCCGT	GCAGCTCAGTAACAGTCCGC

Quantitative real-time PCR reactions were run on a CFX96 Real-time System (Bio-Rad, Hercules, CA). Amplification reactions were performed in quadruplicates, using RT2 SYBR Green qPCR Master mix (QIAGEN, Germany) and 1μM primers in a 10μl volume. All reactions began with initial activation at 95°C for 10min, followed by 45 cycles of 95°C for 15s, 59°C for 20s and 72°C for 15s and a final extension step at 72°C for 7 min. The Ct values were generated by the Bio-Rad CFX Manager software and imported into Microsoft Excel (Microsoft Corporation, Redmond, WA) for conversion to fold expression relative to unit mass (1μg total RNA).

### Histology and Immunochemistry

Transcardiac perfusion was performed first with PBS, then 4% w/v paraformaldehyde and tissues post-fixed overnight. For histological evaluations, tissues were processed for paraffin embedding and sectioned at 7μm for hematoxylin and eosin (HE) staining as described (38). Images were captured with a Nikon Ti Eclipse (Nikon, Japan) and analyzed with the NIS elements software (Nikon). Camera lucida images were generated to map lesion topography. Alternatively, for immunofluorescence staining, brains were cut in the coronal plane immediately caudal to the medial mammillary nucleus, into forebrain and hindbrain blocks, using a mouse brain matrix. Each block was then snap frozen by submerging for 5 s into liquid isopentane cooled to approximately –80°C with dry ice and stored at –80°C prior to sectioning. Brains were sectioned coronally at 30 μm and sections stored in cryoprotectant solution (39). Immunostaining of hippocampal sections was performed as described (17, 40) using polyclonal antibodies to CD3 (at 1:400, Dako, Glostrup, Denmark), ionized calcium-binding adapter molecule 1 ([Iba1] at 1:200, Wako Chemical Industries, Japan), and monoclonal antibodies against MAP2 (at 1:500 Novus Biologicals, Centennial, CO), S1PR1 ([EDG1] at 1:100, OriGene Technologies Inc., Rockford, MD), S1PR3 and S1PR5 ([EDG3 and EDG8], TNF-α and IFN-γ all at 1:100, Novus Biologicals, Abingdon, UK). The specificity of antibody preparations was verified (**Supplementary Table 1** and **Supplementary Figure 2**). Briefly, immunohistochemistry was performed in 24-well tissue culture plates, with n = 4 mice/group x 4 sections/mouse, on an orbital shaker. On day one, sections were twice rinsed in 0.05 M PBS for 15 min, antigen unmasking performed by boiling in tri-sodium citrate (10mM, pH 6.0) for 3 min, followed by an additional 15 min rinse in 0.05 M PBS and pre-incubation in 3% normal goat serum blocking buffer in 0.3% PBST (0.3% v/v Triton X-100, Sigma–Aldrich; in 0.05 M PBS) for 15 min. Sections were then incubated in primary antibody in 0.1% PBST at room temperature overnight. On day two, sections were again rinsed twice in 0.05 M PBS for 15 min, followed by incubation with the appropriate secondary antibody in 0.05 M PBS for 60 min. Detection was achieved with Alexa Fluor 488 or 594 secondary antibodies (Thermo Fisher Scientific, Waltham, MA) and nuclei were stained with 0.001% 4′,6 diamidino-2-phenylindole (DAPI; Sigma Aldrich). Negative controls included (a) absence of primary antibody and (b) substitution of primary antibody with non-immune isotype antibody. Images were acquired (41) with a Zeiss LSM 780 confocal microscope with a 20x objective using the Zen 2011 (Carl Zeiss AG, Oberkochen, Germany) software. For each section, a z-series comprised of 20 images (1024x1024 pixel resolution and 4 averages per image) was used to generate an average projection. Fluorescence intensity (in arbitrary units) was calculated by NIH Image J (http://rsb.info.nih.gov/ij/) and assigned a value between 0 and 255 for each pixel. The average intensity from 2 rectangular regions of interest at equivalent levels of the grey matter was corrected using the intensity measured from equivalent regions of negative controls. The statistical significance of differences between groups was determined by independent samples t-test.

### Statistical Analyses

Behavior parameters were analyzed using Ethovision XT (version 10, Novus Information Technology, Wageningen, The Netherlands) and data analyzed by 2 (treatment:sham *vs*. EAE) X 2 (intervention:PBS *vs*. FTY720) univariate analyses of variance (ANOVA) using the Statistical Package for the Social Sciences (SPSS, v22, IBM, Armonk, NY) for the assessment of group differences between conditions. That is, an interaction effect was examined between the treatment cohort and the intervention cohort, and main effects were also examined. A normality of distribution and homogeneity of variance was assumed and confirmed by a Shapiro–Wilk test (p > 0.05) and Levene’s test (p > 0.05), respectively. Post-hoc tests using Fisher’s protected least significant differences (LSD) were performed for all variables when appropriate. The mean difference is significant at the 0.05 level. The n value was 8 mice/group. All other data were analyzed using a one-way ANOVA and shown as mean ± standard error of the mean (SEM), where the significance level is 0.05.

## Results

### FTY720 Treatment Eliminates Inflammatory Infiltration Along the Neuraxis in EAE-Induced Mice

The human equivalent dose for FTY720 is 0.3mg/kg/day (19), but under experimental conditions in this study this dose resulted in incomplete restoration of lymphocyte counts to normal levels (**Supplementary Figure 3**). Titration of dosage versus lymphocyte counts showed that a minimum of 0.7mg/kg/day was required to restore lymphocyte counts to normal levels. Similarly monocyte counts were significantly reduced with 0.3mg/kg/day, but fully restored to normal levels with 0.7mg/kg/day. Therefore throughout this study we compared the effects of 0.3mg/kg/day (FTY720-L) with 0.7mg/kg/day (FTY720-H).

**Figure 1:**
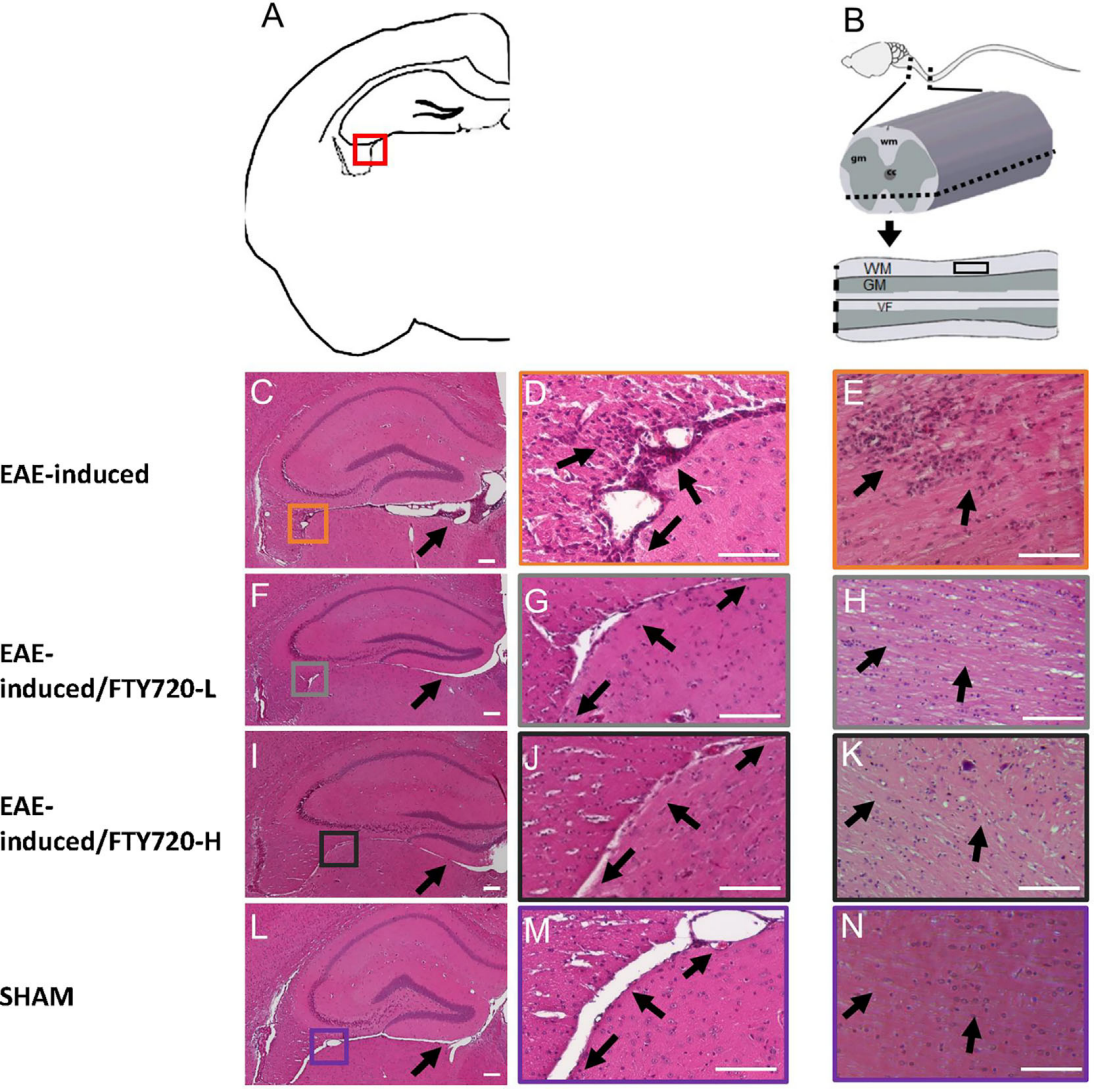
Effect of FTY720 on inflammatory infiltration into the CNS. **(A)** is a schematic of the brain region of interest, with the red box indicating the fimbrium region (also in **C, F, I, L**). All images were taken at 14 dpi. **(C, D)** show representative HE-stained sections from the EAE-induced, untreated group. **(F, G, I, J)** show representative images of the EAE-induced FTY720-treated (F, G = FTY720-L and I, J = FTY720-H) groups and **(L, M)** representative sham sections. **B** is a schematic of the spinal cord region of interest, with the dotted line showing the level of sectioning and the black rectangle the region shown in **(E, H, K, N) E** = EAE-induced, **H** = EAE-induced/FTY720-L, **K** = EAE-induced/FTY720-H, **N** = sham. Scale bar = 150 µm; n = 4 mice/group x 3 repeats.

**Figure 2:**
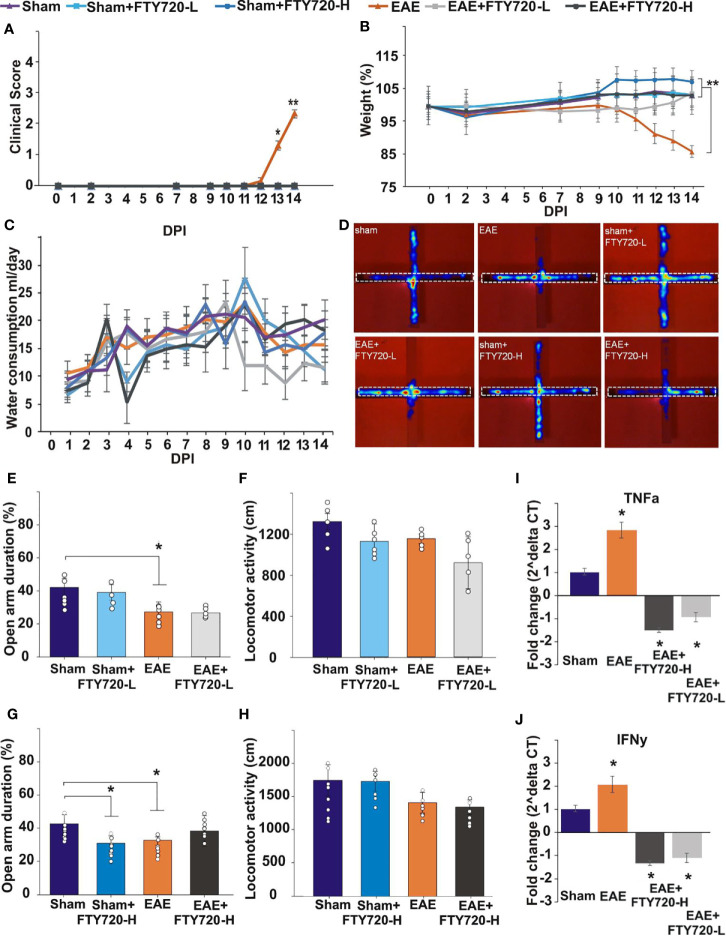
Effect of FTY720 treatment on disease development, anxiety-like behavior, and expression of pro-inflammatory molecules in the hippocampus. **(A, B)** show the disease profile in experimental and control groups, with only the untreated EAE-induced group (p<0.01) exhibiting clinical scores **(A)** and significant weight loss **(B)** by 14 dpi (*p <0.05 and **p <0.01). There was no difference in water consumption between the different groups on the day of the EPM test **(C)**. **(D)** shows the EPM and representative heat maps from each group, with the dotted line showing the closed arm. **(E–H)** show the results of the EPM test at low FTY720 dose, with **(E)** sham (purple), sham+FTY720-L (light blue), EAE (orange) and EAE+FTY720-L groups (light grey) showing no difference between sham and sham+FTY720-L groups, a difference (*) between sham and EAE-induced groups, but no difference between EAE and EAE+FTY720-L groups. **(G)** shows the results conducted at high FTY720 dose in sham (purple), sham+FTY720-H (dark blue), EAE (orange) and EAE+FTY720-H (dark grey) groups. There was no difference between the EAE and EAE+FTY720-H groups, but a difference between the sham and sham+FTY720-H groups (*). There was no significant difference between any of the groups for total distance moved **(F, H)**. **(I, J)** shows the qPCR analysis of pro-inflammatory cytokines TNF-α **(I)** and IFN-γ **(J)** in with significantly elevated TNF-α and IFN-γ in the EAE relative to the sham group and significant reduction (*) of these markers in the EAE+FTY720-L and EAE+FTY720-H groups. In A and B, n = 8 mice/group and in C, n = 4 mice/group and in A-C experiments were replicated 2 times. Non behavior data (A – C, I, J) were analyzed using one-way ANOVA and shown as mean ± standard error of the mean (SEM). Behavior data **(E–H)** were data analyzed by 2 (treatment:sham *vs*. EAE) X 2 (intervention:PBS *vs*. FTY720) ANOVA for the assessment of group differences between conditions. Refer to **Supplementary Tables 2, 3** for further statistical data.

In the untreated EAE-induced group severe inflammation was detected by histological (HE) staining around the hippocampus at experimental end point (**Figure 1**). It was most prominent in the meninges, especially in the region between the ventral aspect of the dorsal hippocampus and the midbrain (**Figure 1C**), as well as within the fimbrium region (**Figure 1D**). Other regions of the hippocampus exhibited minor lesions. On the other hand, no meningeal inflammatory cell accumulation was detected in both EAE+FTY720 treated groups (FTY720-L, **Figures 1F, G**, and FTY720-H, **Figures 1I, J**), nor were any lesions found within the hippocampus itself. Overall, inflammation in EAE-induced and FTY720-treated groups was comparable to that observed in the sham group (**Figures 1L, M**), showing efficacy of FTY720 treatment. The same was true of the spinal cord, namely the observation of florid inflammation predominantly in white matter, in the EAE-induced but not FT720-treated groups (**Figures 1E, H, K, N**). In accordance with these observations, only the EAE-induced group exhibited clinical disease (clinical score of 2.25 ± 0.1; % weight difference in sham *vs* EAE = 13.7 ± 0.15; p<0.01) by 14 dpi, (**Figure 2A**), while the sham, sham+FTY720 treated (both -L or -H), and EAE+FTY720 treated (both -L or -H) groups remained clinical score free, nor did they exhibit weight loss (% weight difference in sham *vs* sham+FTY720-L, EAE+FTY720-L, EAE+FTY720-H = 0; sham *vs* sham+FTY720-H = 3.8 ± 0.5, p>0.05) (**Figure 2B**).

### FTY720 Treatment Did Not Significantly Reduce Anxiety-Like Behavior in EAE-Induced Mice

Anxiety-like behavior was evaluated in the EPM as previously described (33), where deficits are assessed in terms of the percentage of time spent in the open arms of the maze (**Figures 2E, G**, **Supplementary Table 2**). Experimental groups included sham, sham+FTY720 treated (both -L or -H), EAE-induced, and EAE+FTY720 treated (both -L or -H) groups (**Figure 2**) and testing carried out at 9 dpi. The selection of this time point was based on our previous evidence from intracellular cytokine staining identifying the earliest accumulation of autoreactive T cells in blood, lymphoid and CNS tissues (32), as well as histological evidence of earliest parenchymal entry of lymphocytes (42), at 11-12 dpi. Absence of lymphocytic infiltration at 9 dpi was confirmed (**Supplementary Figure 4**). In this experimental series, we examined (a) a potential direct drug effect on anxiety-like behavior, (b) an EAE effect and (c) combined EAE and drug effects. Identification of a potential drug effect in the absence of EAE was performed by comparison of the sham group with sham+FTY720 treated (both -L and -H) groups. There was no significant difference between sham and sham+FTY720-L treated mice (open arm duration %, sham vs. sham+FTY720-L, 39.7 ± 8.0 *vs*. 37.9 ± 7.7, p = 0.305) showing that low dose FTY720 treatment in the absence of EAE is not associated with anxiety. Interestingly, this was not observed in the sham+FTY720-H group where drug treatment resulted in increased anxiety-like behavior, (open arm duration %, sham *vs*. sham+FTY720-H, 40.6 ± 9.0 *vs*. 31.8 ± 4.5, p = 0.043), thereby demonstrating a dose-dependent FTY720 effect on anxiety-like behavior in the absence of EAE.

Determination of an EAE effect on anxiety-like behavior was performed by comparison between the sham and the EAE-induced groups. A significant difference between these groups was demonstrated, confirming our previous observations that EAE induction alone results in anxiety-like behavior (open arm duration %, sham *vs*. EAE, 39.7 ± 8.0 *vs*. 20.4 ± 5.4, p = 0.019) and that this effect is already evident from the preclinical stage (33). Drug effect was then estimated by comparison between EAE and EAE+FTY720 (-L or -H); however, there was no change in the percent time spent in the open arms between the EAE-induced, and either of the EAE+FTY720 treated groups showing no beneficial effect of FTY720 treatment on anxiety-like behavior irrespective of dosage (open arm duration %, EAE vs. EAE+FTY720-L, 20.4 ± 5.4 *vs*. 25.2 ± 1.2, p = 0.442 and EAE *vs*. EAE+FTY720-H, 32.7 ± 7.9 *vs*. 41.2 ± 9.3, p = 0.500).

An additional important measure is that of total distance covered over the time of experimentation to identify potential early ambulatory difficulties, undetectable by visual observation, which may confound the test (**Figures 2F, H**, **Supplementary Table 3**). There was no significant difference in the total distance covered during the test period between groups, including sham groups (distance moved cm, sham vs. sham+FTY720-L, 1758.2 ± 234.0 *vs*. 1743.0 ± 149.6, p = 0.249; sham *vs*. sham+FTY720-H, 1320.6 ± 78.6 *vs*. 1127.5 ± 170.3, p = 0.828), EAE groups (distance moved cm, EAE *vs*. EAE+FTY720-L, 1420.7 ± 155.0 *vs*. 1354.1 ± 109.5, p = 0.604; EAE *vs*. EAE+FTY720-H, 1154.5 ± 46.4 *vs*. 919.8 ± 253.6, p = 0.177) or treatment groups (distance moved cm, sham+FTY720-L *vs*. EAE+FTY720-L, 1743.0 ± 149.6 *vs*. 1354.1 ± 109.5, p = 0.314; sham+FTY720-H *vs*. EAE+FTY720-H, 1127.5 ± 170.3 *vs*. 919.8 ± 253.6, p = 0.842) showing absence of ambulatory difficulties at the time at which experimentation was conducted. At 9 dpi, all mice exhibited a clinical score of zero (**Figure 2A**) as expected and there were no significant differences in percent weight change between groups (weight % from 0 dpi, EAE *vs*. EAE+FTY720-L, 96.9 ± 7.1 *vs*. 98.2 ± 2.8, p = 0.25; EAE *vs*. EAE+FTY720-H, 96.9 ± 7.1 *vs*. 100.1 ± 2.8, p = 0.26) (**Figure 2B**). Finally, monitoring of average water consumption showed that FTY720 was not aversive to the mice and that all groups consumed an average of 4.5 ml/day/mouse (**Figure 2C**).

### FTY720 Treatment Decreased Levels of Pro-Inflammatory Cytokines in EAE-Induced Mice

At 9 dpi, half of the mice in each control group (sham, sham+FTY720 [-L or -H]), and experimental group (EAE and EAE+FTY720 [-L or -H]) were humanely killed and the dorsal hippocampal region collected for total RNA extraction, generation of cDNA and qPCR analysis of the pro-inflammatory cytokines TNF-α and IFN-γ (**Figures 2I, J**). In both cases, comparison of sham and EAE groups revealed a significant difference (p<0.05), showing that even by 9 dpi a severe inflammatory environment was present in the hippocampus. FTY720 treatment resulted in the significant reduction (p<0.05) in expression of the pro-inflammatory markers, as demonstrated by significantly reduced expression levels between EAE+FTY720 (-L or -H) groups and the EAE-induced untreated group. This conclusion was supported by confocal microscopic quantification of TNF-α and IFN-γ levels in the hippocampus (**Supplementary Figure 5**).

### The Pro-Inflammatory Environment in the Hippocampus Is Associated With Sub-Field-Specific Expression of Hallmarks of Neuroinflammation

**Figure 3:**
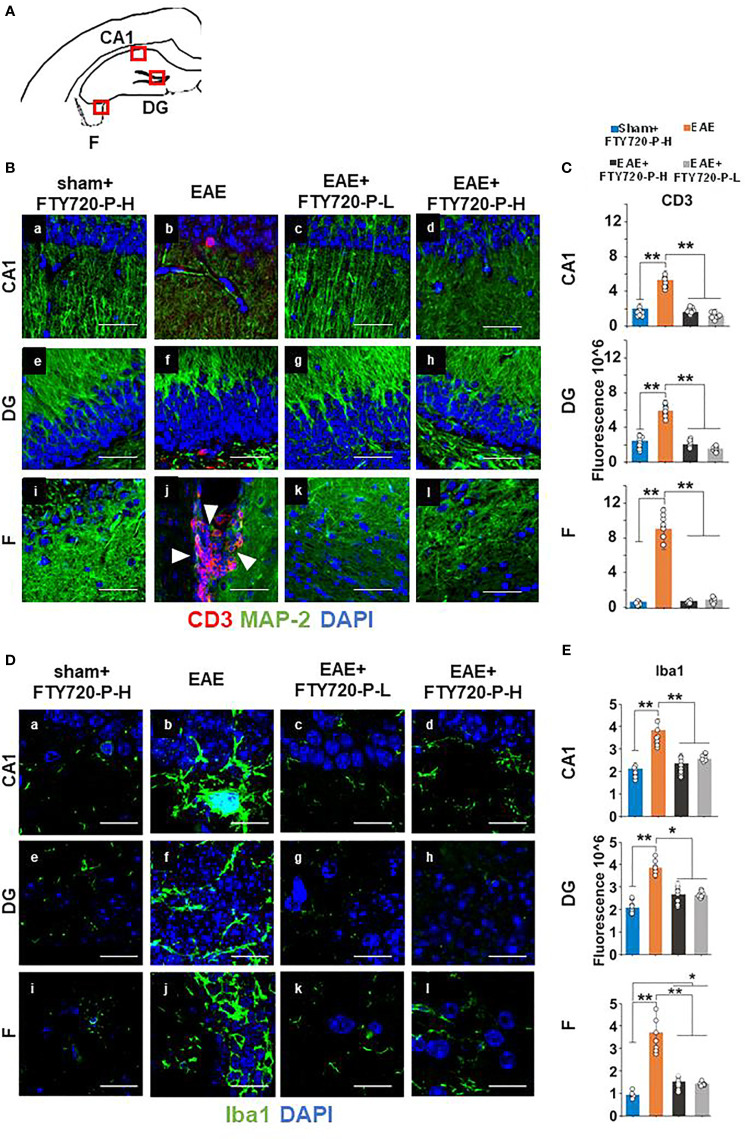
Anti-inflammatory effect of FTY720 on the hippocampus. Following EAE induction, mice received FTY720 *via* drinking water at low dose (EAE+FTY720-L; 0.3mg/kg) or high dose (EAE+FTY720-H; 0.7mg/kg), with *n* = 8 mice/group. Controls consisted of EAE, sham+FTY720-H (shown) and sham+FTY720-L (not shown) groups. Half of each cohort was used to quantify drug effects by immunochemistry in regions shown in **(A)** (red boxes): CA1, dentate gyrus (DG) and fimbrium (F); **(Ba–l)** show anti-CD3 (red) + anti-MAP-2 (green) and **(Da–l),** anti Iba1 (green) immunostaining. Nuclei were stained with DAPI. **(C, E)** show the quantification of CD3, or Iba1 in each region, with sham+FTY720-H (dark blue), EAE (orange), EAE+FTY720-H (dark grey) and EAE+FTY720-L (light grey) groups estimated by ImageJ using an arbitrary scale. N = 4 mice/group × 6 sections/mouse; *p < 0.05, **p < 0.01; scale bars = 20 µm. Experiments were replicated 2 times. Data were analyzed using one-way ANOVA and shown as mean ± standard error of the mean (SEM).

To confirm FTY720 efficacy in mice undergoing the EPM test, quantification of hallmarks of neuroinflammation was performed at 14 dpi in the remaining mice of the cohort. By CD3 immunochemistry (**Figure 3**) no evidence of lymphocytic infiltration was observed in tissues from sham+FTY720-H and EAE+FTY720 (-L and -H) (**Figures 3Ba, c-e, g-i, k, l**). On the other hand, positive staining was present in the EAE-induced group and essentially restricted to the fimbrium and adjacent choroid plexus (**Figures 3Bb, f, j**). These results were quantified, (**Figure 3C**), showing a significant (p<0.01) reduction of infiltrating cells in EAE+FTY720 (-L and -H) groups, particularly in the fimbrium, when compared to the EAE-induced group.

Examination of microglial changes with anti-Iba1 revealed extensive reactivity identified visually by larger cell bodies and more complex branching of processes throughout the whole of the dorsal hippocampus in the EAE-induced group (**Figures 3Db, f, j**). The microglial activity for EAE+FTY720-L (**Figures 3Dc, g, k**), and EAE+FTY720-H (**Figures 3Dd, h, l**) groups appeared reduced relative to the EAE-induced group (**Figures 3Da, De, Di**). These results were quantified, (**Figure 3E**), confirming reduction in microglial reactivity in the EAE-induced+FTY720 (-L and -H) groups relative to EAE-induced (p<0.01). However, this quantification also revealed significant (p<0.05) difference in microglial reactivity between the EAE+FTY720-treated and sham+FTY720-H levels only in the fimbrium. Taken together, these data show that FTY720 is exerting the anticipated effect of reducing hallmarks of neuroinflammation.

### FTY720 Has a Complex Sub-Region-Specific and Dose-Specific Effect on S1PR in the Hippocampus

**Figure 4:**
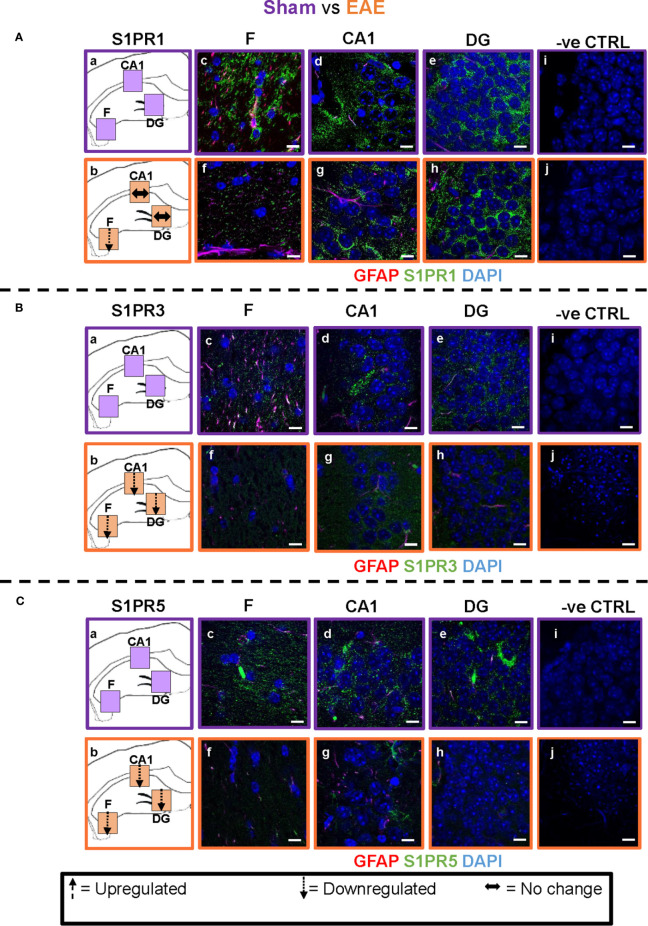
EAE effect on S1PR1, S1PR3 and S1PR5 expression in the hippocampus. Comparison of S1PR expression between sham (purple) and EAE (orange) groups. The left-hand panel shows the regions of interest **(Aa-c, Ba-c, Ca-c)** and the direction of change in three hippocampal sub-fields for each S1PR (downregulation = dotted arrow, upregulation = dashed arrow, or no change = horizontal arrow). The main panel shows immunostained sections with anti-GFAP (red) and anti-S1PR1 (green; **Ac-e, f-h**), anti-S1PR3 (green; **Bc-e, f-h**) or anti-S1PR5 (green; **Cc-e, f-h**); nuclei were stained with DAPI. No primary antibody controls are shown in **Ai-j, Bi-j** and **Ci-j**). Scale bars = 10 µm. Experiments were replicated 3 times. Quantification of images is shown in **Figure 7**.

**Figure 5:**
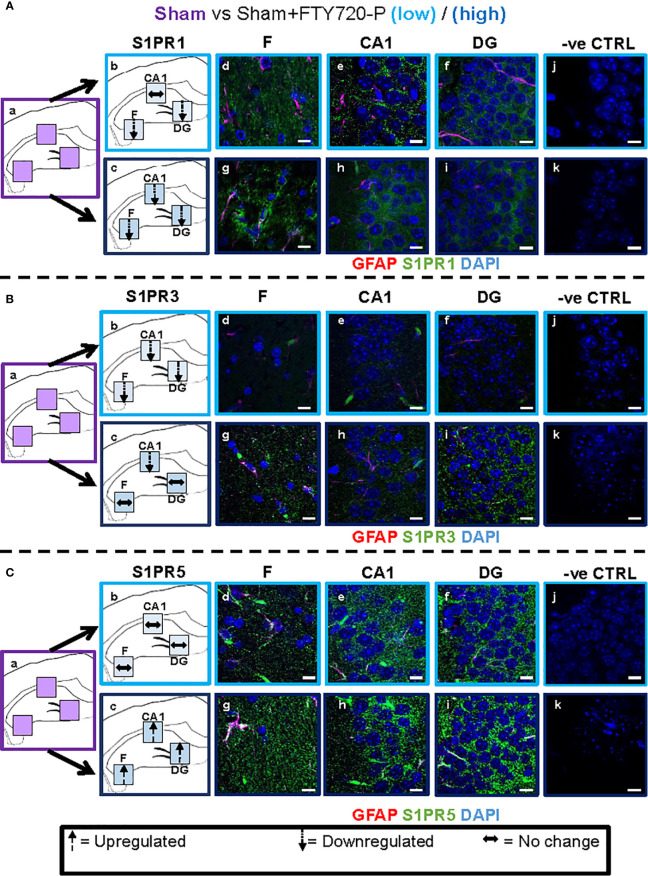
Direct FTY720 effect on S1PR1, S1PR3 and S1PR5 expression in the hippocampus. Comparison of S1PR expression between sham, sham+FTY720-Lo and sham+FTY720-H groups. The left-hand panel shows the regions of interest **(Aa-c, Ba-c, Ca-c)** and the direction of change in three hippocampal sub-fields for each S1PR (downregulation = dotted arrow, upregulation = dashed arrow, or no change = horizontal arrow). The main panel shows immunostained sections with anti-GFAP (red) and anti-S1PR1 (green; **Ad-f, g-i**), anti-S1PR3 (green; **Bd-f, g-i**) or anti-S1PR5 (green; **Cd-f, g-i)**; nuclei were stained with DAPI. Original images for Aa, Ba and Ca are found in **Figure 4**. No primary antibody controls are shown in **Aj-k, Bj-k** and **Cj-k**). *N* = 4 mice/group × 6 sections/mouse. Scale bars = 10 µm. Experiments were replicated 3 times. Quantification of images is shown in **Figure 7**.

**Figure 6:**
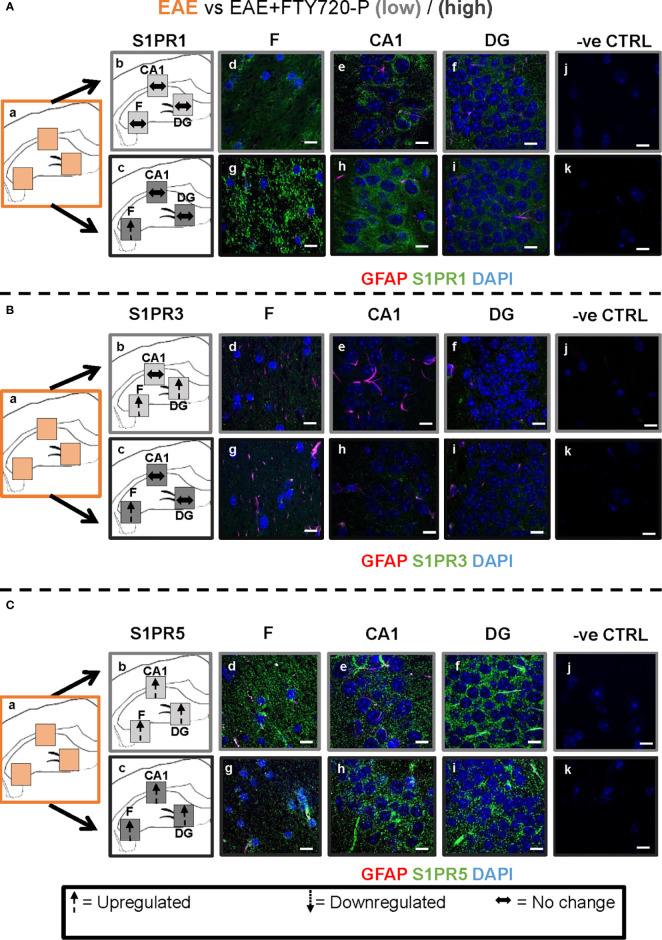
Combined EAE and FTY720 effects on S1PR1, S1PR3 and S1PR5 expression in the hippocampus. Comparison of S1PR expression between EAE, EAE+FTY720-L and EAE+FTY720-H groups. The left-hand panel shows the regions of interest **(Aa-c, Ba-c, Ca-c)** and the direction of change in three hippocampal sub-fields for each S1PR (downregulation = dotted arrow, upregulation = dashed arrow, or no change = horizontal arrow). The main panel shows immunostained sections with anti-GFAP (red) and anti-S1PR1 (green; **Ad-f, g-i**), anti-S1PR3 (green; **Bd-f, g-i**) or anti-S1PR5 (green; **Cd-f, g-i)**; nuclei were stained with DAPI. Original images for Aa, Ba and Ca are found in **Figure 4**. No primary antibody controls are shown in **Aj-k, Bj-k** and **Cj-k**). *N* = 4 mice/group × 6 sections/mouse. Scale bars = 10 µm. Experiments were replicated 3 times. Quantification of images is shown in **Figure 7**.

**Figure 7:**
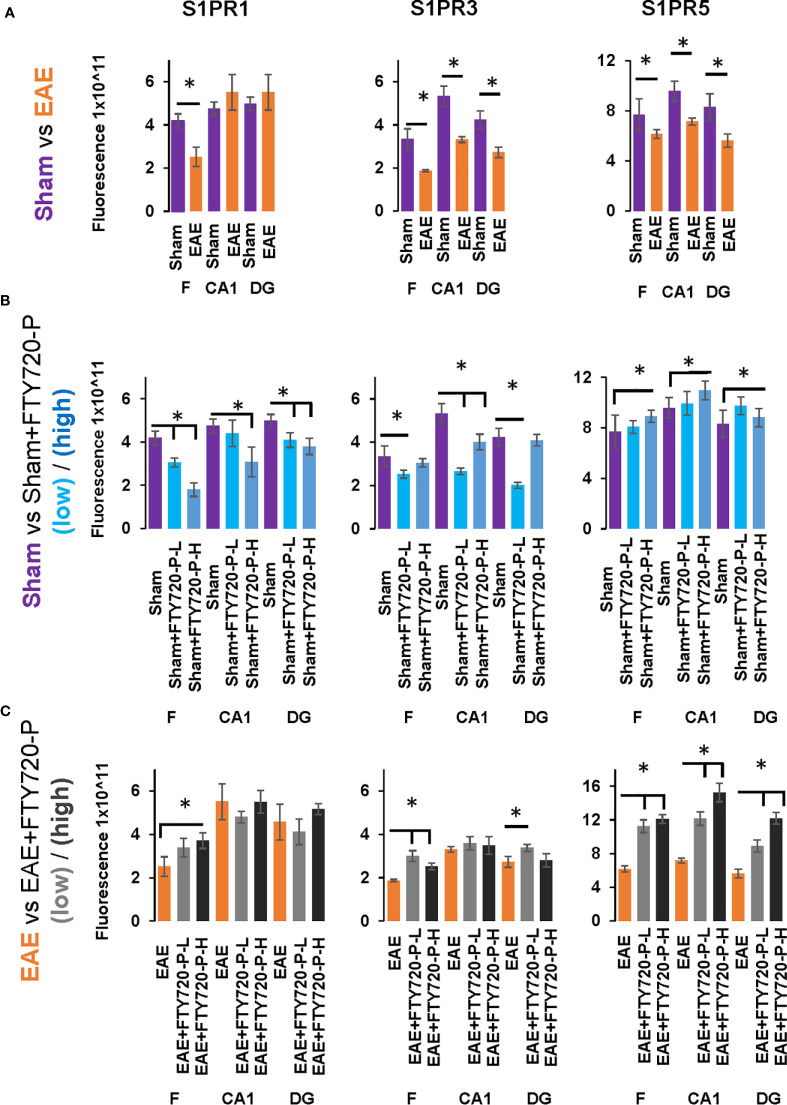
Quantification of S1PR1, S1PR3 and S1PR5 expression in the hippocampus in EAE and following FTY720 treatment. **(A)** shows the EAE effect (EAE *vs* sham, images from **Figure 4**) from each hippocampal sub-region, with sham (purple) and EAE (orange), with signals captured using an arbitrary scale and estimated by ImageJ. **(B)** shows the direct FTY720 effect on sham mice (sham *vs* sham+FTY720, images from **Figure 5**) on S1PR1, S1PR3 and S1PR5 expression in the hippocampus with sham (purple), sham+FTY720-L (light blue) and sham+FTY720-H (dark blue). **(C)** shows the combined EAE and FTY720 effects (EAE *vs* EAE+FTY720, images from **Figure 6**) on S1PR1, S1PR3 and S1PR5 expression in the hippocampus with EAE (orange), EAE+FTY720-L (light grey) and EAE+FTY720-H (dark grey). *N* = 4 mice/group × 6 sections/mouse (*p<0.05). Experiments were replicated 3 times. Data were analyzed using one-way ANOVA and shown as mean ± standard error of the mean (SEM).

The evidence of direct FTY720 effect on hippocampal function was further explored by immunochemical quantification of expression of the known S1PR in the dorsal hippocampus at 9 dpi. FTY720 has been shown to recognize four of the five known receptors, S1PR2, being the exception (43–46). Additionally, S1PR4 is very poorly expressed in the adult CNS (43). QPCR analysis of RNA extracted from hippocampus biopsies confirmed expression of only S1PR1, S1PR3 and S1PR5 in this region (**Supplementary Figure 6**). Therefore, quantification of only these three members of the S1PR family was performed and was restricted to the fimbrium, CA1 region and the dentate gyrus. Results showed complex changes which were receptor-specific, sub-field specific and dose-specific (**Figures 4**–**7** and **Supplementary Table 4**).

#### EAE Effect

Comparison of EAE versus sham groups showed downregulation of S1PR1 in the fimbrium (p<0.01) and no change in the CA1 and dentate gyrus (**Figures 4A** and **7**) (p>0.05). S1PR3 and S1PR5 both exhibited downregulation (**Figures 4B, C** and **7**) (p<0.01).

#### Direct FTY720 Effect

A direct effect of the drug on the hippocampus was confirmed by comparison of receptor expression in sham mice, versus sham+FTY720-L and sham+FTY720-H (**Figures 5** and **7**). S1PR1 showed downregulation in the fimbrium and the dentate gyrus, but not the CA1 region at low dose, with downregulation in all three regions at high dose (**Figures 5A** and **7**) (p<0.01). S1PR3 showed downregulation in all three regions at low dose; whereas at high dose this trend was partially reversed in the fimbrium and the dentate gyrus (p<0.01), but not the CA1 region (**Figures 5B** and **7**) (p>0.05). S1PR5 showed no change at low dose (p>0.05), but strong upregulation at high dose (**Figures 5C** and **7**) (p<0.01). The net result of FTY720 on the hippocampus is an effect in opposite directions for S1PR1 and S1PR5 expression, but absence of direct relationship between dosage and expression levels for S1PR3.

#### FTY720 Effects on EAE

Comparison of EAE versus EAE+FTY720-L or H was made to identify drug effect upon EAE induction (**Figures 6** and **7**). At low dose, there was no change in S1PR1 in any of the three regions of interest (**Figures 6A** and **7**) (p>0.05), S1PR3 showed upregulation in the fimbrium and dentate gyrus (p<0.01), but no change in the CA1 region (**Figure 6B** and **7**) (p>0.05) and S1PR5 showed strong upregulation in all three regions (**Figures 6C** and **7**) (p<0.05). With increasing dosage, S1PR1 and S1PR3 were upregulated in the fimbrium alone, while remaining unchanged in the other two regions and S1PR5 was upregulated across all three regions. Therefore, the overall trends were for no change in S1PR1 with increasing dosage, except for an increase in the fimbrium. S1PR3 showed upregulation then downregulation and S1PR5 consistent upregulation from low to high dose.

Other additional conclusions that can be made from these observations are that (a) the effects of the drug upon EAE induction are not additive, namely effects seen in the presence of each of these conditions can be reversed upon combination, (b) S1PR1, S1PR3 and S1PR5 in the fimbrium region tended to be increasingly upregulated with increasing FTY720 dose, (c) S1PR1 and S1PR3 in the CA1 region exhibited no response to the drug, irrespective of dose; on the other hand S1PR5 in the same region was strongly upregulated from low dose and (d) S1PR1 in the dentate gyrus did not respond to the drug, while S1PR5 in the same region strongly responded to it; on the other hand S1PR3 showed opposing trends with strong upregulation at low dose and no change at high dose.

## Discussion

### Anxiety-Like Behavior Is Evident From the Preclinical Stage in EAE

The aim of this investigation was to obtain evidence for a primary disease mechanism underlying the major psychiatric sequelae of multiple sclerosis, namely anxiety and major depression, *via* behavioral assays in EAE. Because of the involvement of the hippocampus (a major target region in MS; estr) in these symptoms and of the proposed role of proinflammatory cytokines in the manifestation of depression, we characterized pathological changes in the hippocampus and effects of immunomodulation on lymphocytic infiltration and behavior. In earlier studies, we validated EAE for investigations of hippocampal pathology by the demonstration that EAE hippocampal changes essentially recapitulate those observed in MS, whereby tissue destruction is associated with minor infiltration in the grey matter subfields, but with severe inflammation in the fimbrium, a white matter subfield, as well as in the surrounding meninges [**Figure 1** and (17)]. Additionally, we identified the earliest evidence of autoreactive T cells in blood, lymphoid tissues and the CNS parenchyma between 10 and 12 dpi (33). Here using the EPM, a validated experimental paradigm for rodent-specific anxiety-like behavior, we show that significant deficits are already evident by 9 dpi, associated with a highly inflammatory environment, as demonstrated by elevated proinflammatory cytokines and microglial reactivity (**Figure 2**). Thus, altered hippocampal function begins in the preclinical stage, prior to detectable autoreactive T cells. This would be presumably due to soluble inflammatory factors originating from innate immune cells and platelets, entering the hippocampus from the meninges and/or *via* regions devoid of BBB, for example around the peri-ventricular aspects. This would result in triggering microglial reactivity and the generation of further pathogenic cascades.

Thus, the early evidence of anxiety-like behavior in EAE provides proof of concept for a primary neurodegenerative component underlying depression associated with MS. However, our evidence also shows that these symptoms precede lymphocytic infiltration and suggest that their earliest manifestation probably coincides with early accumulation of pro-inflammatory mediators Originating From Innate Immune Cells.

### Immunomodulation Has No Beneficial Effect on Anxiety-Like Behavior

To target inflammation, mice were treated with FTY720. The efficacy of this immunomodulator was demonstrated by absence of EAE development (**Figures 1** and **2**), confirmed by lack of detectable lesions in the spinal cord and hippocampus by experimental end point, together with significant reduction in expression of hallmarks of neuroinflammation in the hippocampus, such as CD3 positive cell infiltration and microglial reactivity (**Figures 2** and **3**). It is also notable that significant reduction of pro-inflammatory cytokines was evident by 9 dpi. This reduction is presumably attributable to an effect of FTY720 on innate immune cells, since effects of this drug beyond their established ones on lymphocyte trafficking have been demonstrated. For example it is known that FTY720 reduces the mobility of dendritic cells by downregulation of adhesion molecules mediating endothelial adhesion and transmigration, thereby promoting their retention in the circulation. FTY720 has also been associated with an impaired innate immune response in gastrointestinal infection, manifested by significantly reduced dendritic cells and macrophages and reduced macrophage infiltration.

Despite its efficacy in reducing inflammation, FTY720 was not beneficial in reducing anxiety-like behavior in EAE-induced mice. This was evidenced (**Figure 2**) by the absence of significant difference between the EAE and both EAE+FTY720 groups, in the EPM test, thereby showing that increasing dosage did not result in improvement of deficits. This lack of treatment effect is not attributable to ambulatory difficulties, because testing was performed during the preclinical stage and estimation of total locomotor activity showed no significant difference between any of the experimental and control groups. Previously (47), showed a significant effect of FTY720 in the light-dark box test in EAE at a low dose, equivalent to that used in this study. These different conclusions may be related to variations in experimental design between the two studies. In our hands, a more severe disease profile is generated, therefore the EPM test was performed at 9 dpi, corresponding to clinical score of zero, to avoid potential confounding effects caused by ambulatory difficulties. In the Bonfiglio study a milder disease progression was evident and testing was performed at 18 dpi, namely after sustained treatment. However, taken together, the data may also suggest that the drug may be of benefit in the presence of disease of moderate severity, but not when disease progression is aggressive.

On the other hand, while FTY720 exhibited no significant effect on anxiety-like behavior in EAE-induced mice, an anxiogenic effect of the drug was identified (**Figure 2**). This was revealed by absence of significant difference in time spent in the open arms of the maze between the sham and sham+FTY720-L groups, but significant difference between the same groups at high dose. This may be a result of direct interactions between the drug and S1PR, since due to its lipophilic nature, FTY720 has the capacity to cross the blood brain barrier (43). Our data showed expression of S1PR1, S1PR3 and S1PR5 in the adult hippocampus. Moreover, FTY720 has also been found to have additional molecular targets and functions (44, 48–50), which may directly impact on the EPM test at high dose. In this context, differential effects of FTY720 between low and high doses on hippocampal function have also been demonstrated in a murine model of epilepsy (51). Treatment at low dose (0.3 mg/kg) reduced the frequency of seizures and epileptic form discharges, while high dose (1 mg/kg) had no effect.

### FTY720 Has Direct Effects on S1PR in EAE

To explore the potential differential effects of EAE and FTY720 on the dynamics of S1PR, expression of each of the three receptors of interest was evaluated by quantitative confocal microscopy at both high and low dose. Three sub-fields were selected because they are functionally distinct (9) and their well-defined morphology facilitates reproducibility of sampling. To our knowledge, no similar comprehensive study of S1PR modulation by FTY720 in relation to hippocampal function has been described (42, 43). Complex responses were identified, varying with EAE induction, FTY720 dosage, specific receptor and sub-region used in quantification (**Figures 4**–**7**), but evaluation of S1PR changes in expression levels with respect to evidence, or otherwise, of anxiety-like behavior suggest some trends (**Supplementary Table 4**).

- Effects of FTY720 alone at low dose ranged from no change to decrease of the three receptors of interest, which did not result in behavioral change. At high dose, while decrease was associated with S1PR1 or no change with S1PR3, a highly significant increase was observed in S1PR5 expression. Therefore anxiety-like behavior resulting from high dose FTY720 is associated principally with increased S1PR5 expression and to a lesser extent with decrease of S1PR1.
- EAE induction was associated with overall decrease in receptor expression, particularly S1PR3 and S1PR5. This observation concurs partly with the literature which reports alterations in S1PR expression levels in the spinal cord in rat EAE, but differs in that S1PR1 and S1PR5 were found to be downregulated, while S1PR3 and S1PR4 were upregulated (52). These differences illustrate region-specific differences in response to FTY720, by S1PR.
- Combined EAE+FTY720 resulted in different effects on the three receptors of interest. In the case of S1PR1 and S1PR3, reverse effects relative to EAE alone, or FTY720 alone were observed and the net effect was that of no change. However, S1PR5 exhibited the most noticeable change, being highly significantly upregulated at both low and high doses, in all fields examined. Since both EAE+FTY720L and EAE+FTY720H combinations are anxiogenic, it can be concluded that there is an association between anxiety-like behavior and upregulation of S1PR5. Also of interest is the observation that in addition to S1PR5, S1PR1 and S1PR3 were almost consistently upregulated in the fimbrium region. The fimbrium contains both efferent and afferent fibers travelling between the dorsal hippocampus and fornix, a white matter tract connecting the hippocampus to the hypothalamus, which represent two major stress-regulatory regions of the CNS (9). Therefore, upregulation of the three receptors of interest in the fimbrium is potentially of high functional significance.

Overall, these data suggest that FTY720 effects are mediated more strongly *via* S1PR5 in the CA1 and DG sub-fields, but *via* S1PR1, S1PR3 and S1PR5 in the fimbrium. The involvement of S1PR5 is consistent with the predominant CNS expression of this member of the S1PR family (53), but also suggests immediate effects of FTY720 on this receptor. In trying to interpret these data, it is important to remember that the S1PR are distinct subtypes, that regulate a wide range of fundamental biological processes, as well as immunomodulation (43). Each of these receptors has a unique signature in terms of cellular expression, response to S1P signaling and range of downstream cascades. Neurons, oligodendrocytes and their precursor populations, astrocytes, microglia and endothelial cells all express S1PR at various levels and the relationships between these neural cells differ between subfields, for example by absence on neuronal cell bodies in the fimbrium. Additionally, FTY720 modulates the receptors of interest differentially (26, 45, 54). Furthermore, only three sub-fields of the hippocampus were surveyed for S1PR expression, suggesting even greater complexity across the whole structure. It is therefore unsurprising that no direct relationship between drug dosage, S1PR expression and anxiety-like behavior can be identified.

Finally, an additional observation was that, while FTY720-L alone is not anxiogenic, the combination of EAE and FTY720-L is. Dosages between 0.1 mg/kg body weight to over 0.1 mg/kg are used in the literature, but 0.3 mg/kg is the human equivalent dosage. Therefore at a clinically relevant dose the drug appears to be anxiogenic on EAE.

### Clinical Implications of Findings

Depression in MS is now an active area of investigation (20, 55, 56). Trials conducted to determine the effect of disease modifying treatments on these MS symptoms have revealed no increased risk of adverse psychiatric events with FTY720 and similarly, treatment of MS patients with antidepressants appears to be somewhat beneficial (4, 57). This may seem to be in contradiction to present results; however, the effects of treatments have been consistently shown to be partial. Most of the widely used antidepressant medications act by reuptake inhibition of neurotransmitters at the synaptic cleft, particularly serotonin. Selective serotonin reuptake inhibitors have additional anti-inflammatory (particularly TNF-α induced inflammation) and anti-oxidative effects, thereby shifting the pro-inflammatory environment towards homeostatic levels (58). It is important to remember that in MS patients the beneficial effects of disease modifying therapies or antidepressants on anxiety and depression symptoms will also result from indirect contribution of additional factors, such as a more positive outlook on the control of disease activity, or increased social interactions for example. Therefore, evidence presented here, of a pro-inflammatory environment in the hippocampus prior to the accumulation of autoreactive T cells in EAE, with consequences on function, is critical. It provides proof of concept for a mechanism underlying depressive symptoms prior to neurological deficits in MS. Additionally, data showed that at clinically relevant dosage, FTY720 is anxiogenic in EAE, principally *via* S1PR5. However, these adverse effects may not be associated with new-generation S1PR modulators which are characterized by higher selectivity for S1PR1. The implications therefore are that (a) early monitoring for anxiety and depression must become part of MS management to identify contributions from primary mechanisms, which may be masked by those resulting from psychosocial causes and (b) more research is required to develop therapeutics specifically targeting mood disorders in MS.

## Data Availability Statement

The datasets presented in this article are not readily available because a proportion of the data were generated under a contract with Novartis Pharmaceuticals and is confidential. Requests to access the datasets should be directed to J.Orian@latrobe.edu.au.

## Ethics Statement

The animal study was reviewed and approved by La Trobe University Animal Ethics Committee.

## Author Contributions

JO and MH designed the study. PK, NT-S, SM and PD conducted experiments and acquired data. JO, MH, PK and NT-S analyzed data. JO, PK, NT-S and MH wrote the manuscript. All authors contributed to the article and approved the submitted version.

## Funding

This research was funded in part by Novartis Pharmaceuticals. PK is the recipient of an Australian Postgraduate Award from the Australian Government Department of Education and Training.

## Conflict of Interest

JO received funding from Novartis Pharmaceuticals for partial support of the project and conference attendance.

The funders had no role in study design, data collection and analysis, or preparation of the manuscript.

The remaining authors declare that the research was conducted in the absence of any commercial or financial relationships that could be construed as a potential conflict of interest.

## Supplementary Material

Supplementary Figure 1 Experimental design. Mice were randomly assigned to the following groups: EAE+FTY720-L, EAE+FTY720-H, sham+FTY720-L and sham+FTY720-H mice and healthy controls. FTY720 treatment was initiated at 7 dpi either at low dose (0.3 mg/kg body weight), or high dose (0.7 mg/kg) and the EPM test performed at 9 dpi on the EAE+FTY720-L, EAE+FTY720-H, sham+FTY720-L and sham+FTY720-H mice groups. The EPM test was performed for 5 minutes, following which half of the mice in each group were killed for either cryostat sectioning and quantitative immunochemical evaluation of S1PR1, S1PR3 and S1PR5, or total RNA extraction and qPCR evaluation of TNFα and IFNγ. Of the remaining mice, half were killed at 12 dpi to determine the relationship between dosage and restoration of lymphocyte and monocyte counts to normal levels. Finally, the last animals were killed at 14 dpi for cryostat sectioning and quantitative immunochemical evaluation of markers of neuroinflammation. The box on the right-hand side shows treatment effect on performance in the EPM test, with absence of anxiety-like behavior only in the sham+FTY720-L group, despite drug efficacy. The bottom panel shows the relationship between the innate and adaptive immune cell accumulation and that, given our evidence of earliest detectable autoreactive T cells at 12 dpi (33) anxiety-like behavior detected at 9dpi coincides with the presence of innate rather than adaptive immune cells.

Supplementary Figure 2 Immunochemical analysis of anti-S1PR1, anti-S1PR3 and anti-S1PR5 specificity on neonatal and adult tissues. Receptor expression of S1PR1, S1PR3 and S1PR5 was mapped in a range of neonatal and adult tissues by qPCR and results summarized in **Supplementary Table 1**. Triplicate sections from selected tissues identified as either positive or negative for each receptor were challenged with anti-S1PR1 **(A–D)**, or anti-S1PR3 **(E–H)** or anti-S1PR5 **(I–L)** and detected with the appropriate AlexaFluor 488-conjugated secondary antibody. Blood vessels were stained with anti-albumin, detected with AlexaFluor 594-conjugated secondary antibody and nuclei were stained with DAPI, as described in Materials and Methods. Images were captured as described. For each tissue and each antibody preparation a negative control in the form of absence of primary antibody (Ab) was performed concurrently. Scale bars = 20 µm.

Supplementary Figure 3 Lymphocyte and monocyte counts in normal, EAE, EAE+FTY720L and EAE+FTY720H. To evaluate the effects of FTY720 treatment on disease development one cohort was taken at 12 dpi, which corresponds to the time when lymphocytes become detectable in blood, lymphoid tissues and the CNS, together with clinical onset in EAE mice. Blood was drawn into a K2EDTA coated blood microtainer (Becton-Dickinson (BD), Franklin Lakes, NJ, USA). The microtainer was shaken vigorously to ensure that the sample was completely mixed with the anticoagulant. Samples were stored at room temperature prior to carrying out the total blood counts (TBC). TBCs were determined on an automated haematology analyser, Sysmex XS-1000i (Sysmex America Inc., Mundelein, IL, USA). Blood samples were diluted 1 in 10 with PBS and three readings taken per sample for accuracy. There was a significant difference in lymphocyte counts between EAE mice vs control (normal mice) (7.3±0.8 x 10^3^ vs 3.8±0.3 x 10^3^, p = 0.019), EAE vs EAE+FTY720H (7.3±0.8 x 10^3^ vs 3.9±0.2 x 10^3^, p = 0.015), EAE+FTY720L vs EAE+FTY720H (5.3±0.3 x 10^3^ vs 3.9±0.2 x 10^3^, p = 0.001), normal vs EAE+FTY720L (3.8±0.3 x 10^3^ vs 5.3±0.3 x 10^3^, p = 0.039) groups, but no significant difference between EAE vs EAE+FTY720L (7.3±0.8 x 10^3^ vs 5.3±0.3 x 10^3^, p = 0.08), or normal and EAE+FTY720H (3.9±0.2 x 10^3^ vs 3.9±0.2 x 10^3^, p = 0.77) groups. This shows that the dose of 0.7 mg/kg was required to reduce lymphocyte counts to normal levels by the time corresponding to clinical onset, under conditions used. The n value was 6 mice/group. Data were analyzed using a two-tailed Student’s t-test and shown as mean ± standard error of the mean (SEM). Monocyte counts were estimated similarly. There was a significant difference between EAE mice vs control (normal mice) (0.24±0.04 x 10^3^ vs 0.12±0.01 x 10^3^, p = 0.04), EAE vs EAE+FTY720H (0.24±0.04 x 10^3^ vs 0.12±0.01 x 10^3^, p = 0.04) and EAE vs EAE+FTY720L (0.24±0.04 x 10^3^ vs 0.15±0.03 x 10^3^, p = 0.05) groups, but no significant difference between normal vs EAE+FTY720L (0.12±0.01 x 103 vs 0.15±0.03 x 10^3^, p = 0.52), normal vs EAE+FTY720H (0.12±0.01 x 10^3^ vs 0.12±0.1 x 10^3^, p = 0.6), or EAE+FTY720L vs EAE+FTY720H (0.15±0.03 x 10^3^ vs 0.12±0.01 x 10^3^, p = 0.6) groups. This shows that the dose of 0.3 mg/kg was sufficient to reduce monocyte counts to normal levels by the time corresponding to clinical onset, under conditions used.

Supplementary Figure 4 Absence of inflammatory infiltration along the neuraxis, at 9 dpi. The entire CNS was dissected from mice at 9 dpi and prepared for histology, as described under ‘*Materials and Methods*’. Images **(A–N)** are all representative images from 9 dpi mice, with n = 3. Sections were cut at 7 µm at multiple levels along the neuraxis and HE stained. Regions shown in yellow boxes in **(B, D, G, I, K, M)** were selected based on our experience with spatio-temporal lesion development in EAE (17, 32, 33, 36, 38, 40, 42). They indicate white matter regions where lesions will be reproducibly found in over 95% of EAE-induced animals, from 11-12 dpi onwards. Here, by contrast, at 9 dpi no evidence of inflammatory infiltration is found in these regions. Similarly, arrows in E indicate white matter regions devoid of inflammation at 9 dpi, corresponding to the region shown in **Figures 1D, G, J, M** where prominent inflammation was observed by 14 dpi. These data support our evidence that altered hippocampal function as identified by the EPM test at 9 dpi, precedes lymphocytic infiltration. Scale bar = 150 µm, except for **(A–C)** (7 µm) and **(F–H)** (70 µm).

Supplementary Figure 5 Expression of TNF-α and IFN-γ in the hippocampus and effect of FTY720 treatment. Mice from the sham group, EAE and EAE+FTY720 [-L or -H]) were humanely killed and tissues prepared for immunochemistry with antibodies to cytokines TNF-α **(A)** and IFN-γ **(B)**. Quantitative confocal microscopy was performed in the CA1 region of the dorsal hippocampus. In the case of TNF-α, a strong effect of the drug is observed at both low and high doses; in the case of IFN-γ, significant drug effect is observed at high dose only.

Supplementary Figure 6 Effect of EAE induction on S1PR expression in the hippocampus. Experimental conditions and primers for qPCR analysis of S1PR1 S1PR2, S1PR3, S1PR4 and S1PR5 are described in the Materials and Methods section (**Table 1**). Expression of S1PR1, S1PR3, and S1PR5, was detected in EAE-induced (orange) mice, but was negative for S1PR2 and S1PR4 following normalization against sham mice; hence analysis of only S1PR1, S1PR3, and S1PR5 at the protein level. FTY720 treatment was associated with significantly reduced levels of S1PR1, S1PR3, and S1PR5 expression at both dosages relative to the EAE group. Experiments were replicated 3 times, n = 4 mice/group. Data were analyzed using a two-tailed Student’s t-test and shown as mean ± standard error of the mean (SEM).
