# Supplementary figures and images for: Immunomodulation Eliminates Inflammation in the Hippocampus in Experimental Autoimmune Encephalomyelitis, but Does Not Ameliorate Anxiety-Like Behavior
Source: Front Immunol. 2021 Jun 10;12:639650. doi: 10.3389/fimmu.2021.639650 (PMC8222726; doi:10.3389/fimmu.2021.639650)

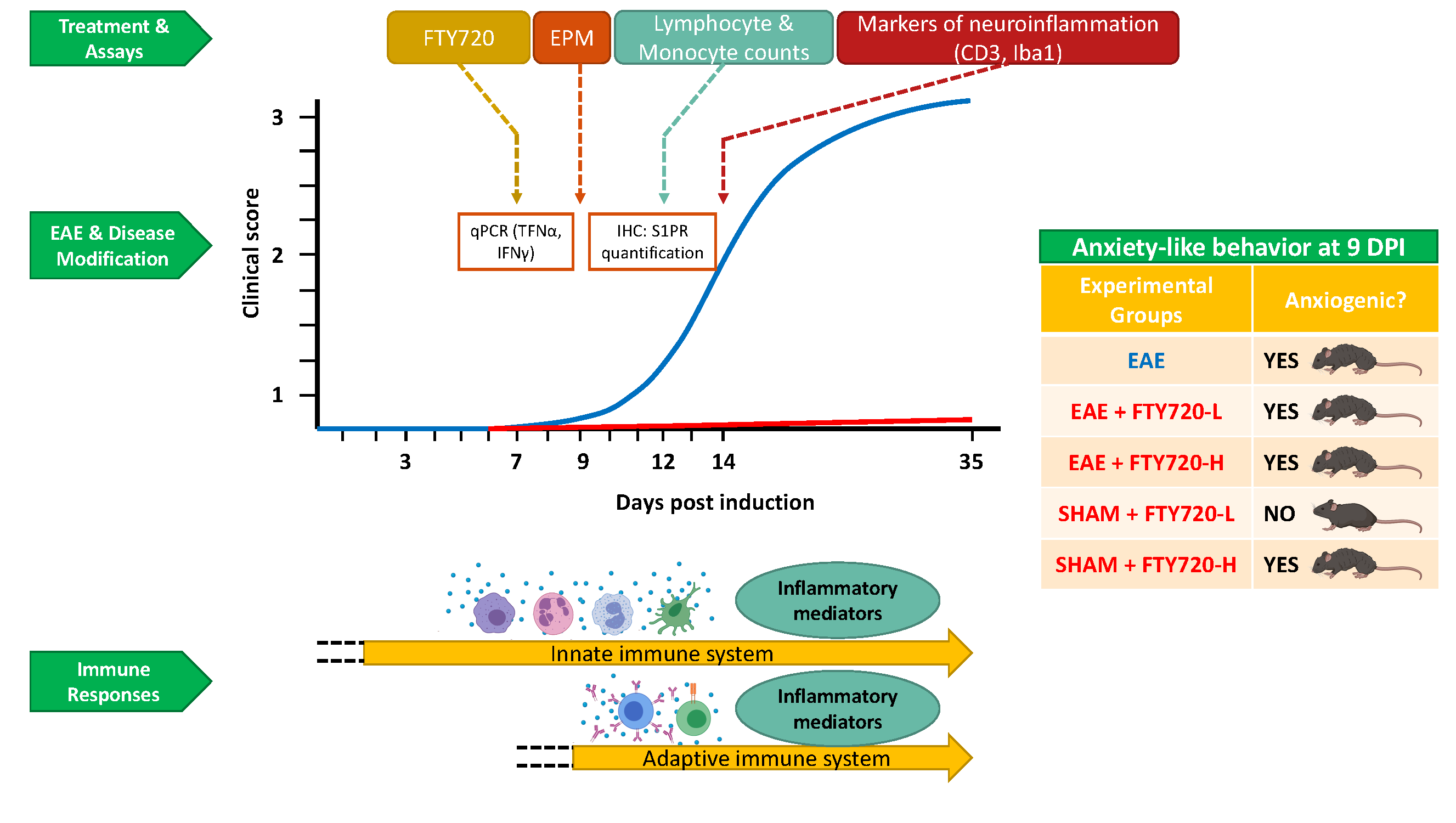

Supplement: Supplementary Figure 1 — Experimental design. Mice were randomly assigned to the following groups: EAE+FTY720-L, EAE+FTY720-H, sham+FTY720-L and sham+FTY720-H mice and healthy controls. FTY720 treatment was initiated at 7 dpi either at low dose (0.3 mg/kg body weight), or high dose (0.7 mg/kg) and the EPM test performed at 9 dpi on the EAE+FTY720-L, EAE+FTY720-H, sham+FTY720-L and sham+FTY720-H mice groups. The EPM test was performed for 5 minutes, following which half of the mice in each group were killed for either cryostat sectioning and quantitative immunochemical evaluation of S1PR1, S1PR3 and S1PR5, or total RNA extraction and qPCR evaluation of TNFα and IFNγ. Of the remaining mice, half were killed at 12 dpi to determine the relationship between dosage and restoration of lymphocyte and monocyte counts to normal levels. Finally, the last animals were killed at 14 dpi for cryostat sectioning and quantitative immunochemical evaluation of markers of neuroinflammation. The box on the right-hand side shows treatment effect on performance in the EPM test, with absence of anxiety-like behavior only in the sham+FTY720-L group, despite drug efficacy. The bottom panel shows the relationship between the innate and adaptive immune cell accumulation and that, given our evidence of earliest detectable autoreactive T cells at 12 dpi (33) anxiety-like behavior detected at 9dpi coincides with the presence of innate rather than adaptive immune cells. [file Image_1.tif]

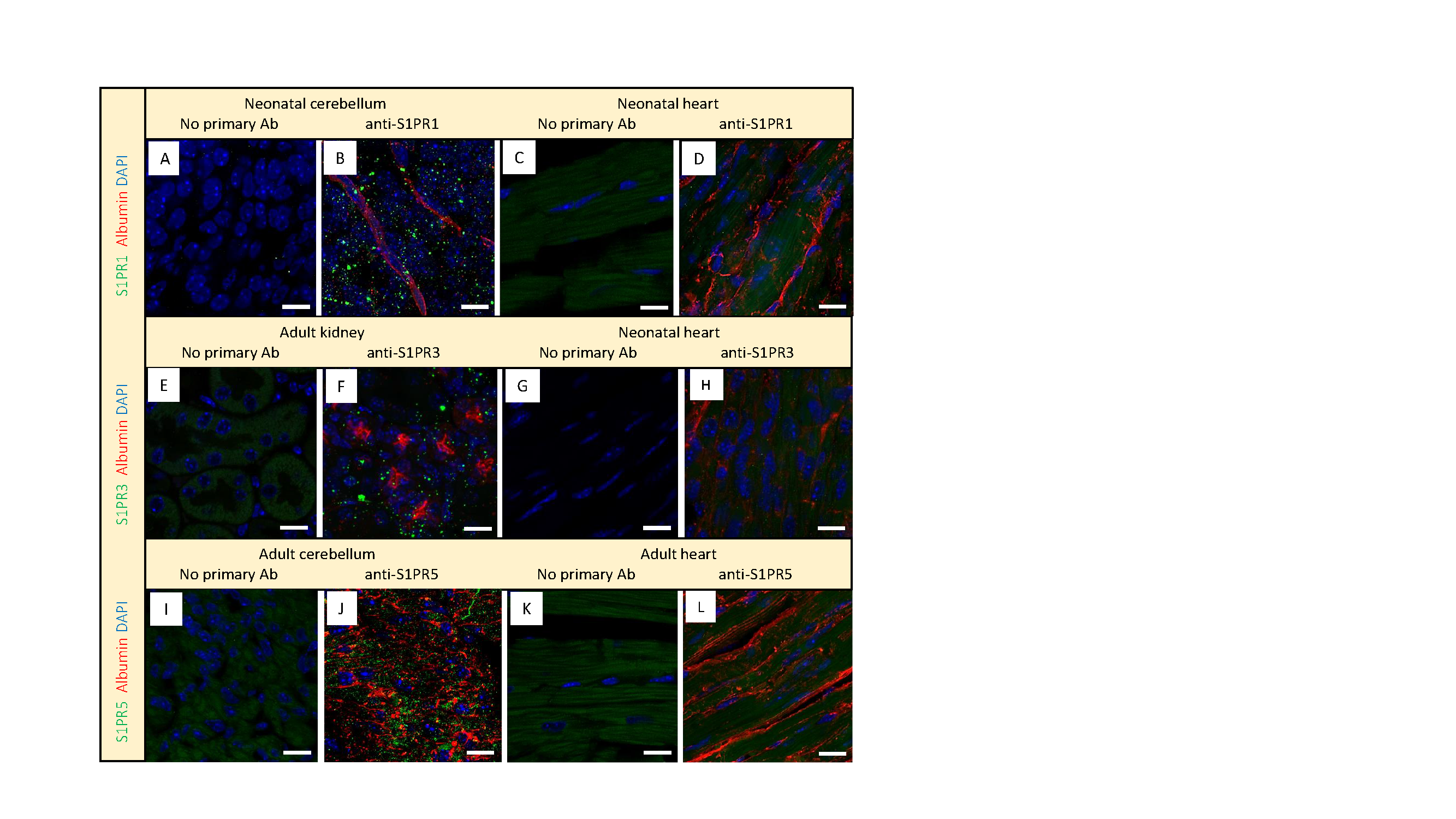

Supplement: Supplementary Figure 2 — Immunochemical analysis of anti-S1PR1, anti-S1PR3 and anti-S1PR5 specificity on neonatal and adult tissues. Receptor expression of S1PR1, S1PR3 and S1PR5 was mapped in a range of neonatal and adult tissues by qPCR and results summarized in Supplementary Table 1 . Triplicate sections from selected tissues identified as either positive or negative for each receptor were challenged with anti-S1PR1 (A–D), or anti-S1PR3 (E–H) or anti-S1PR5 (I–L) and detected with the appropriate AlexaFluor 488-conjugated secondary antibody. Blood vessels were stained with anti-albumin, detected with AlexaFluor 594-conjugated secondary antibody and nuclei were stained with DAPI, as described in Materials and Methods. Images were captured as described. For each tissue and each antibody preparation a negative control in the form of absence of primary antibody (Ab) was performed concurrently. Scale bars = 20 µm. [file Image_2.tif]

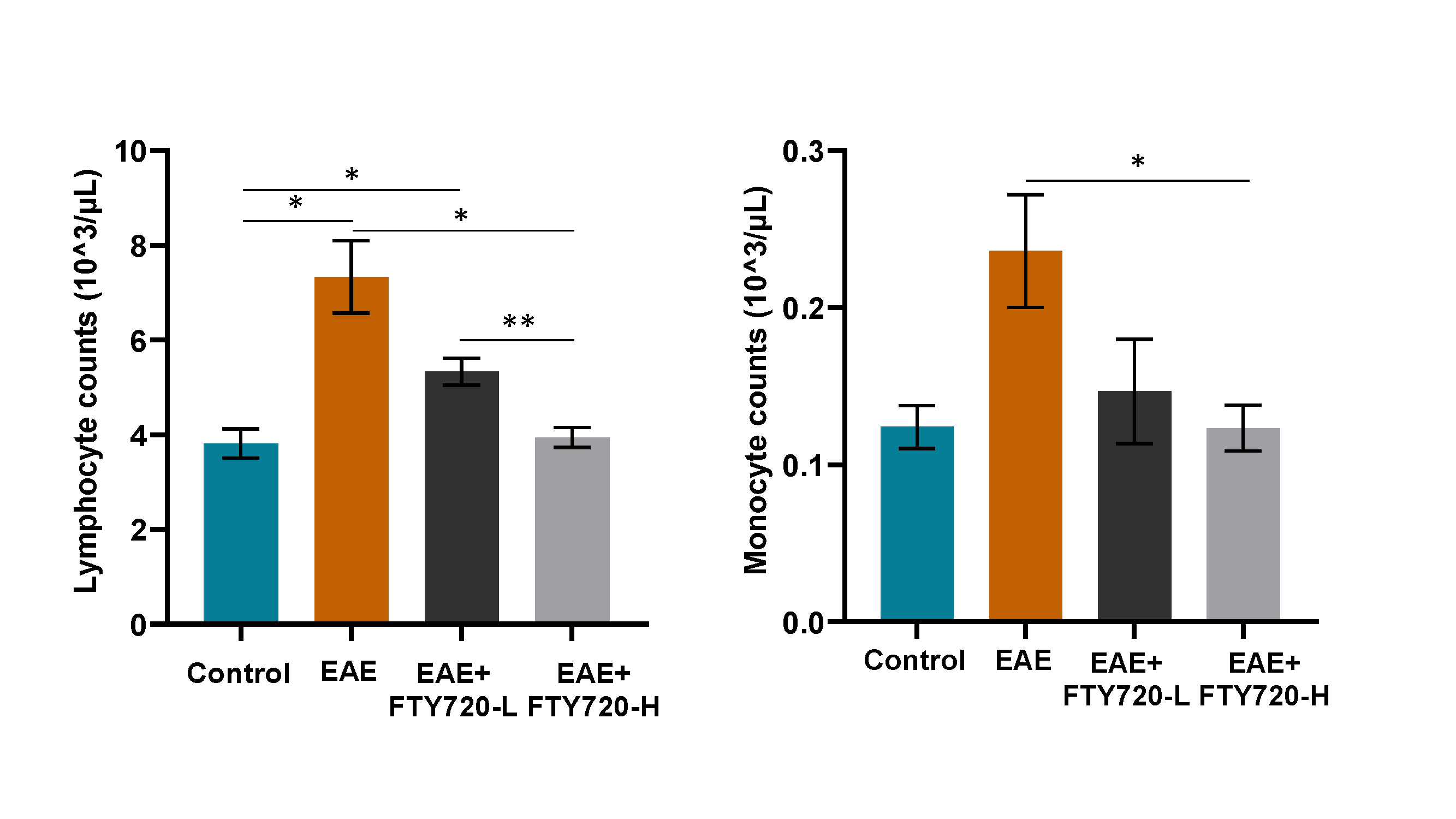

Supplement: Supplementary Figure 3 — Lymphocyte and monocyte counts in normal, EAE, EAE+FTY720L and EAE+FTY720H. To evaluate the effects of FTY720 treatment on disease development one cohort was taken at 12 dpi, which corresponds to the time when lymphocytes become detectable in blood, lymphoid tissues and the CNS, together with clinical onset in EAE mice. Blood was drawn into a K2EDTA coated blood microtainer (Becton-Dickinson (BD), Franklin Lakes, NJ, USA). The microtainer was shaken vigorously to ensure that the sample was completely mixed with the anticoagulant. Samples were stored at room temperature prior to carrying out the total blood counts (TBC). TBCs were determined on an automated haematology analyser, Sysmex XS-1000i (Sysmex America Inc., Mundelein, IL, USA). Blood samples were diluted 1 in 10 with PBS and three readings taken per sample for accuracy. There was a significant difference in lymphocyte counts between EAE mice vs control (normal mice) (7.3±0.8 x 10^3^ vs 3.8±0.3 x 10^3^, p = 0.019), EAE vs EAE+FTY720H (7.3±0.8 x 10^3^ vs 3.9±0.2 x 10^3^, p = 0.015), EAE+FTY720L vs EAE+FTY720H (5.3±0.3 x 10^3^ vs 3.9±0.2 x 10^3^, p = 0.001), normal vs EAE+FTY720L (3.8±0.3 x 10^3^ vs 5.3±0.3 x 10^3^, p = 0.039) groups, but no significant difference between EAE vs EAE+FTY720L (7.3±0.8 x 10^3^ vs 5.3±0.3 x 10^3^, p = 0.08), or normal and EAE+FTY720H (3.9±0.2 x 10^3^ vs 3.9±0.2 x 10^3^, p = 0.77) groups. This shows that the dose of 0.7 mg/kg was required to reduce lymphocyte counts to normal levels by the time corresponding to clinical onset, under conditions used. The n value was 6 mice/group. Data were analyzed using a two-tailed Student’s t-test and shown as mean ± standard error of the mean (SEM). Monocyte counts were estimated similarly. There was a significant difference between EAE mice vs control (normal mice) (0.24±0.04 x 10^3^ vs 0.12±0.01 x 10^3^, p = 0.04), EAE vs EAE+FTY720H (0.24±0.04 x 10^3^ vs 0.12±0.01 x 10^3^, p = 0.04) and EAE vs EAE+FTY720L (0.24±0.04 x 10^3^ vs 0.15±0.03 x 10^3^, p = 0.05) groups, but no signific [file Image_3.tif]

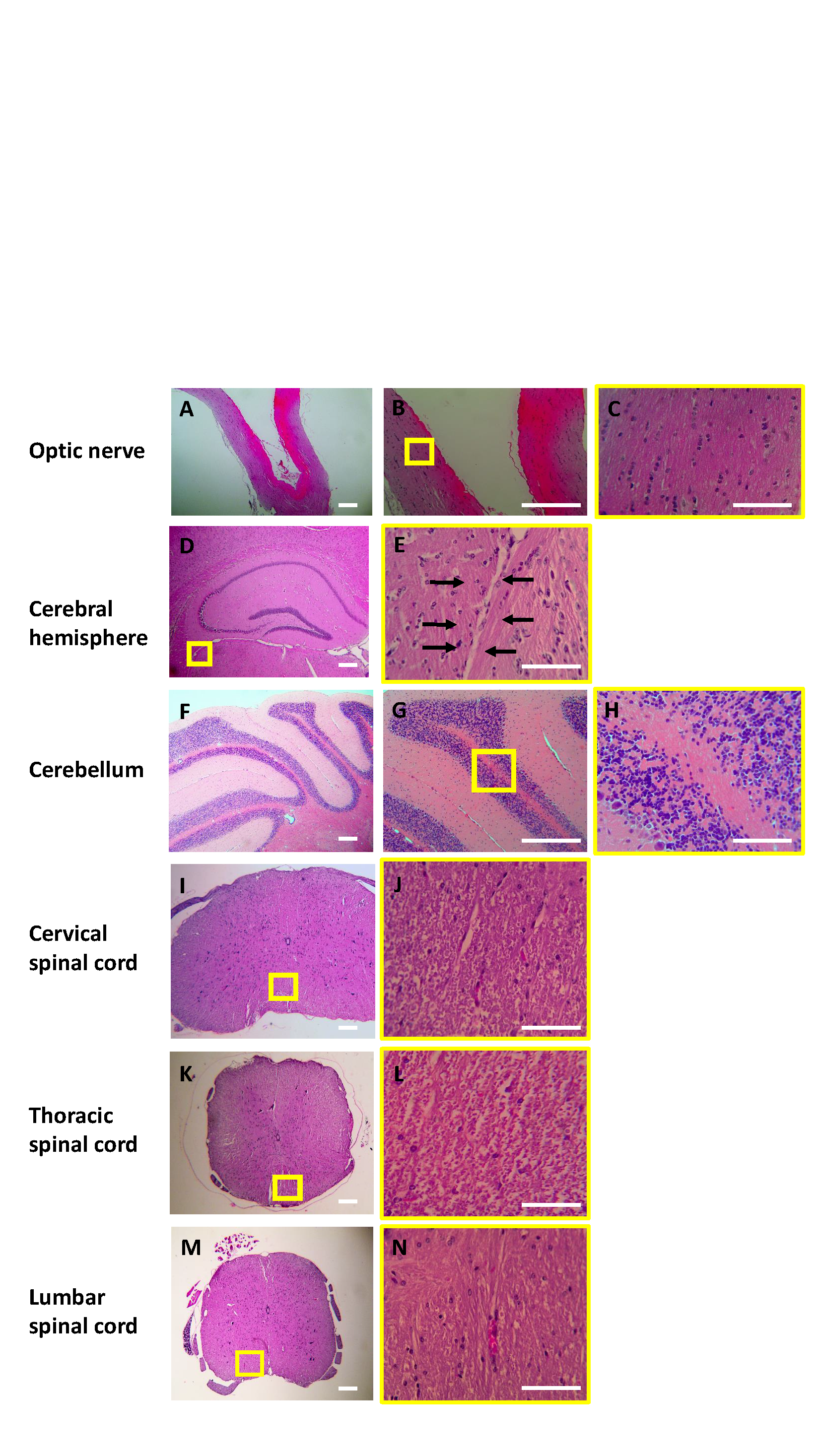

Supplement: Supplementary Figure 4 — Absence of inflammatory infiltration along the neuraxis, at 9 dpi. The entire CNS was dissected from mice at 9 dpi and prepared for histology, as described under ‘Materials and Methods’. Images (A–N) are all representative images from 9 dpi mice, with n = 3. Sections were cut at 7 µm at multiple levels along the neuraxis and HE stained. Regions shown in yellow boxes in (B, D, G, I, K, M) were selected based on our experience with spatio-temporal lesion development in EAE (17, 32, 33, 36, 38, 40, 42). They indicate white matter regions where lesions will be reproducibly found in over 95% of EAE-induced animals, from 11-12 dpi onwards. Here, by contrast, at 9 dpi no evidence of inflammatory infiltration is found in these regions. Similarly, arrows in E indicate white matter regions devoid of inflammation at 9 dpi, corresponding to the region shown in Figures 1D, G, J, M where prominent inflammation was observed by 14 dpi. These data support our evidence that altered hippocampal function as identified by the EPM test at 9 dpi, precedes lymphocytic infiltration. Scale bar = 150 µm, except for (A–C) (7 µm) and (F–H) (70 µm). [file Image_4.tif]

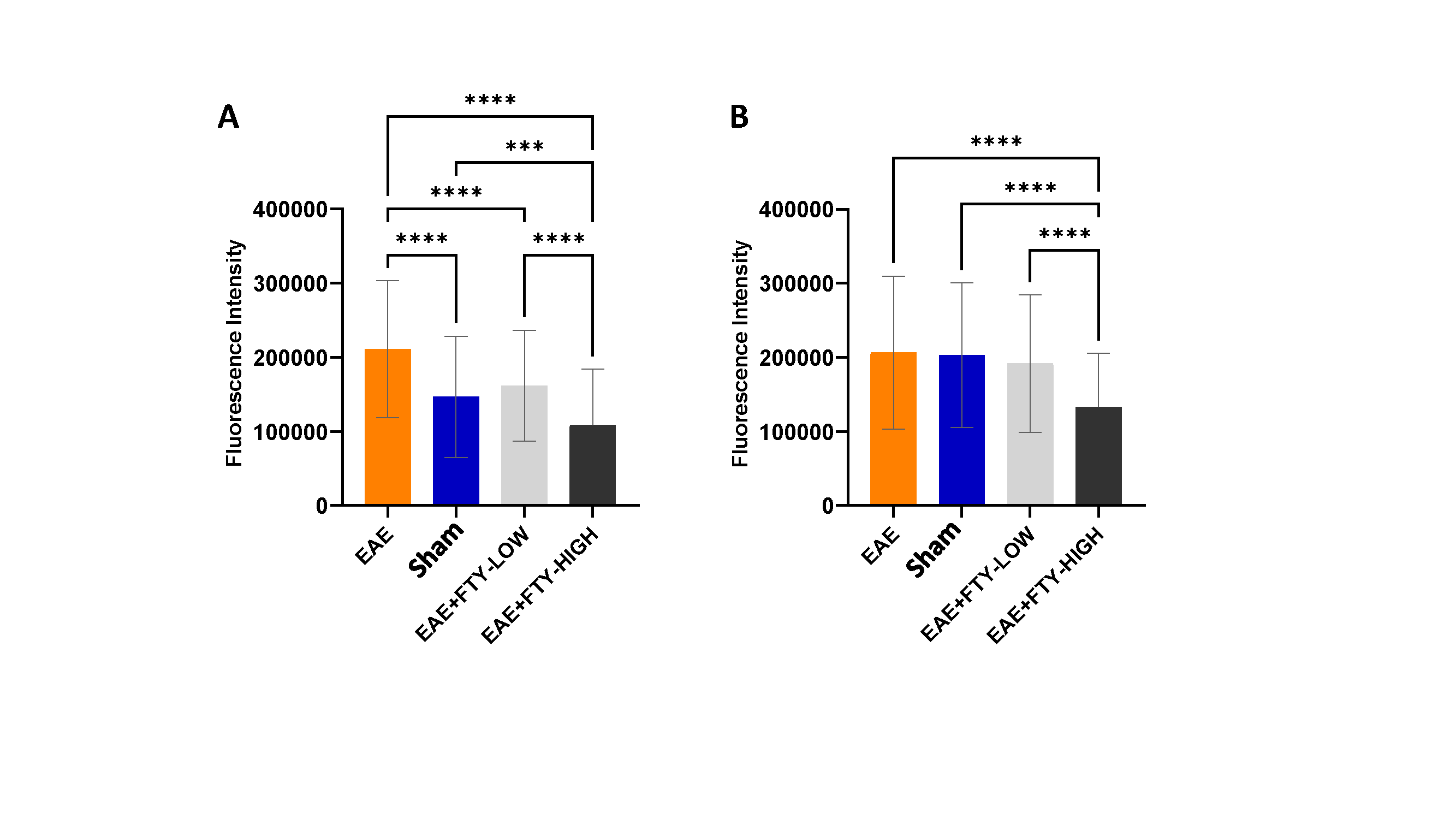

Supplement: Supplementary Figure 5 — Expression of TNF-α and IFN-γ in the hippocampus and effect of FTY720 treatment. Mice from the sham group, EAE and EAE+FTY720 [-L or -H]) were humanely killed and tissues prepared for immunochemistry with antibodies to cytokines TNF-α (A) and IFN-γ (B). Quantitative confocal microscopy was performed in the CA1 region of the dorsal hippocampus. In the case of TNF-α, a strong effect of the drug is observed at both low and high doses; in the case of IFN-γ, significant drug effect is observed at high dose only. [file Image_5.tif]

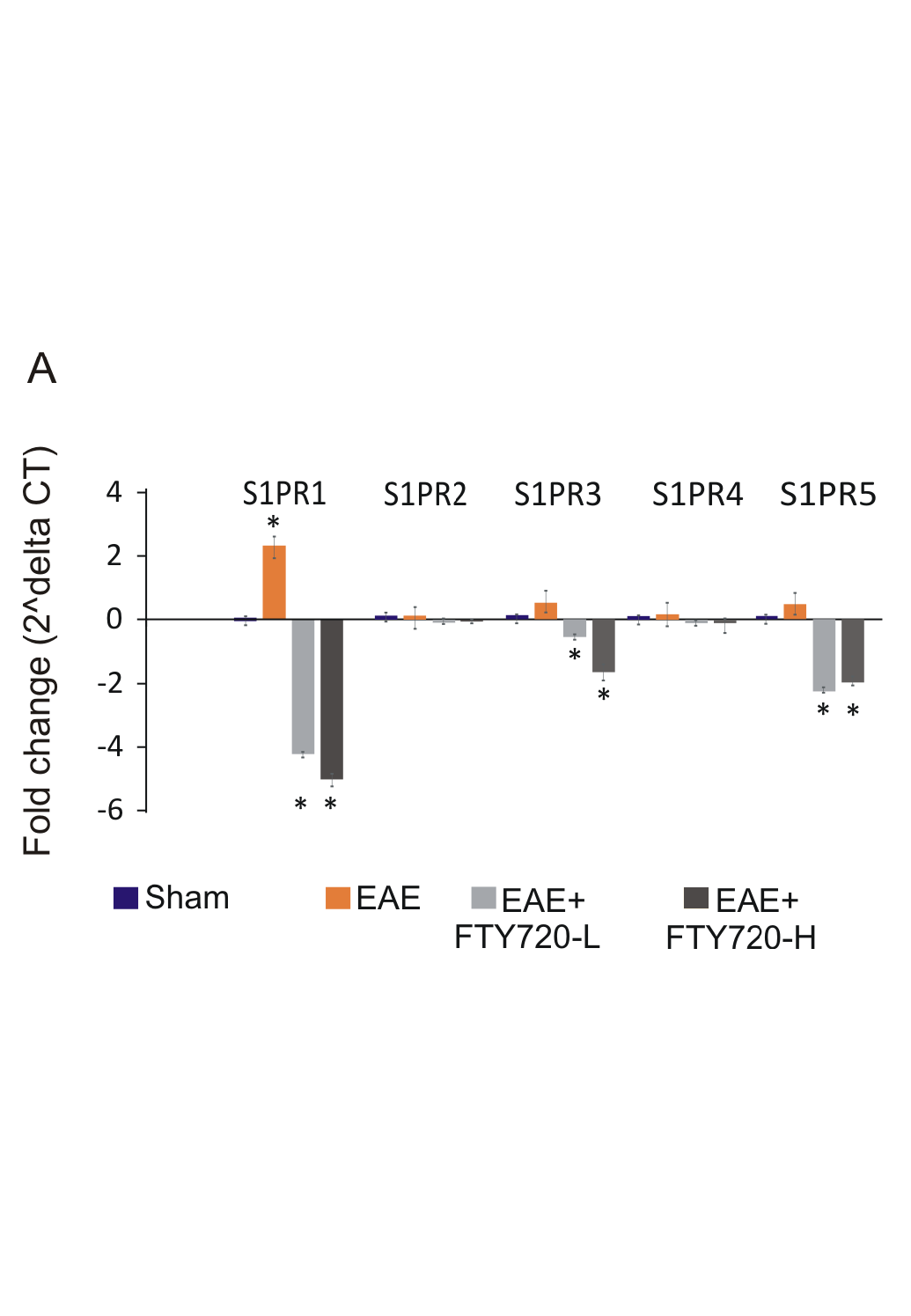

Supplement: Supplementary Figure 6 — Effect of EAE induction on S1PR expression in the hippocampus. Experimental conditions and primers for qPCR analysis of S1PR1 S1PR2, S1PR3, S1PR4 and S1PR5 are described in the Materials and Methods section ( Table 1 ). Expression of S1PR1, S1PR3, and S1PR5, was detected in EAE-induced (orange) mice, but was negative for S1PR2 and S1PR4 following normalization against sham mice; hence analysis of only S1PR1, S1PR3, and S1PR5 at the protein level. FTY720 treatment was associated with significantly reduced levels of S1PR1, S1PR3, and S1PR5 expression at both dosages relative to the EAE group. Experiments were replicated 3 times, n = 4 mice/group. Data were analyzed using a two-tailed Student’s t-test and shown as mean ± standard error of the mean (SEM). [file Image_6.tif]
