# Supplementary material for: Immunomodulation Eliminates Inflammation in the Hippocampus in Experimental Autoimmune Encephalomyelitis, but Does Not Ameliorate Anxiety-Like Behavior
Source: Front Immunol. 2021 Jun 10;12:639650. doi: 10.3389/fimmu.2021.639650 (PMC8222726; doi:10.3389/fimmu.2021.639650)
Supplement: Supplementary file 7 [file DataSheet_1.docx]

**Supplementary Tables 1-3**

**Supplementary Table 1. Verification of specificity of antibodies to S1PR1, S1PR3 and S1PR5 by qPCR and immunochemistry.**

Antibody specificity was ascertained by verification of reactivity on normal mouse tissues, previously identified as negative or positive for expression of S1PR1, S1PR3 and S1PR5 by qPCR. Thus, expression was first determined by screening a range of tissues from neonatal and adult mouse (as per Methods section), transcardially perfused with DEPC treated ice-cold PBS. Most tissues exhibited expression of all three receptors from early post-natal stage (Table 1), but distinct differences in expression levels between neonatal and adult stages were noted in some tissues. Negative tissues for S1PR1 included neonatal brain and heart. Neonatal brain, cerebellum and heart were negative for S1PR3 and neonatal and adult heart and skin were negative for S1PR5. Subsequently, expression of each receptor was determined in a subset of tissues by immunochemistry, against both negative and positive tissues for each antibody preparation (as per Methods section). Data showed a 100 percent concordance between protein level and RNA expression. Representative examples are shown in supplementary image 1, where S1PR1 shows abundant presence by immunochemistry in the neonatal cerebellum, but is undetectable in neonatal heart tissue sections, S1PR3 expression is high in adult kidney, but negative in neonatal heart and S1PR5 is negative in the neonate heart, but positive in adult cerebellum.

**Supplementary Table 1. Identification of tissues exhibiting either positive or negative for expression of S1PR1, S1PR3 and S1PR5.**

| **Developmental stage** | **Retina** | **Brain** | **Cerebellum** | **Heart** | **Lung** | **Liver** | **Kidney** | **Spleen** | **Skin** | **Receptor** |
| --- | --- | --- | --- | --- | --- | --- | --- | --- | --- | --- |
| Neonate | + | - | ++ | - | + | ++ | ++ | nd | + | S1PR1 |
| Adult | ++ | ++ | ++ | + | ++ | ++ | ++ | ++ | + |  |
| Neonate | ++ | - | - | - | ++ | ++ | + | nd | ++ | S1PR3 |
| Adult | ++ | ++ | ++ | + | ++ | ++ | ++ | ++ | ++ |  |
| Neonate | ++ | ++ | ++ | - | + | ++ | + | nd | - | S1PR5 |
| Adult | + | ++ | ++ | - | + | + | + | ++ | ++ |  |

Tissues were collected from normal neonatal mice, aged 4 days and from normal adult mice and processed for total RNA extraction, generation of cDNA and qPCR evaluation of S1PR1, S1PR3 and S1PR5, as described (see Methods section). Expression levels were identified as negative (-), moderate (+) where the fold change was ≤ 20, or high where the fold change was ≥ 20; nd = not determined; n = 3 mice/tissue/antibody.

**Supplementary Table 2. The main effects and interaction effects of the 2 parameters under investigation, on the percentage of total time spent in open arms of the EPM**. The main effects (induction and treatment) and interactions between these parameters are shown in terms of percent open arm duration in a 2 x 2 Univariate ANOVA. Statistical difference (p≤0.05) is found for the treatment effect.

| Percent open arm duration (FTY720-H) | Percent open arm duration (FTY720-L) | 2X2 Univariate ANOVA | |
| --- | --- | --- | --- |
| F(1,28) = 4.663, p = 0.040 | F(1,19) = 6.669, p = 0.018 | Induction | Main effects |
| F(1,28) = 0.043, p = 0.837 | F(1,19) = 0.061 p = 0.808 | Treatment |  |
| F(1,28) = 1.625, p = 0.213 | F(1,19) = 0.283, p = 0.601 | Induction x Treatment | Interaction effect |

**Supplementary Table 3. The main effects and interaction effects of the 2 parameters under investigation, on locomotor activity**. The main effects (induction and treatment) and interactions between these parameters are shown in terms of distance moved (cm) in a 2 x 2 Univariate ANOVA. No statistical difference (p≥0.05) is found for the treatment effect.

| Distance moved (cm) (FTY720-H) | Distance moved (cm) (FTY720-L) | 2X2 Univariate ANOVA | |
| --- | --- | --- | --- |
| F(1,28) = 0.023, p = 0.879 | F(1,20) = 0.661, p = 0.426 | Induction | Main effects |
| F(1,28) = 0.059, p = 0.810 | F(1,20) = 0.661 p = 0.426 | Treatment |  |
| F(1,28) = 0.023, p = 0.879 | F(1,20) = 0.005, p = 0.946 | Induction x Treatment | Interaction effect |

**Supplementary Table 4. Summary of changes in expression of S1PR1, S1PR3 and S1PR5 following EAE induction and treatment with FTY720.** Data were compiled from quantitative confocal microscopic evaluation of expression of each receptor from cryostat sections (30 µm thick), following evaluation of anxiety-like behavior in the EPM test. (* p<-0.05, ** p<0.01, *** p<0.001). Crossed-out boxes show absence of anxiolytic effect.

| Receptor | FTY720 effect  (sham vs FTY720-L)  (sham vs FTY720-H) | EAE effect  (EAE vs sham) | EAE+FTY720 effects  (EAE vs EAE+FTY720-L)  (EAE vs EAE+FTY720-H) | Overall |
| --- | --- | --- | --- | --- |
| S1PR1 | **Low dose**:  Fimbrium – **decrease****  CA1 – **no change** DG – **decrease*** | Fimbrium – **decrease****  CA1 – **no change**  DG – **no change** | **Low dose**:  Fimbrium – **no change**  CA1 – **no change** DG – **no change** | Direct effect of FTY720 at low dose (except CA1 region) and high dose. EAE affects S1PR1 expression levels differentially in the sub-fields of interest. In the presence of EAE, FY720 has no detectable effect at low or high dose (except for the fimbrium at high dose). |
|  | **High dose**:  Fimbrium – **decrease***** CA1 – **decrease*** DG – **decrease*** |  | **High dose**:  Fimbrium – **increase*** CA1 – **no change** DG – **no change** |  |
| S1PR3 | **Low dose**:  Fimbrium – **decrease*** CA1 – **decrease***** DG – **decrease***** | Fimbrium – **decrease****  CA1 – **decrease*****  DG – **decrease***** | **Low dose**:  Fimbrium – **increase***** CA1 – **no change** DG – **increase*** | Direct effect of FTY720 at low dose, reversed in CA1 and Fimbrium at high dose. EAE decreases S1PR3 expression levels. In the presence of EAE, FTY720 upregulates S1PR3 levels differentially in the 3 sub-fields of interest. |
|  | **High dose**:  Fimbrium - **no change**  CA1 – **decrease**** DG – **no change** |  | **High dose**:  Fimbrium – **increase*****  CA1 – **no change** DG – **no change** |  |
| S1PR5 | **Low dose**:  Fimbrium - **no change** CA1 – **no change** DG – **no change** | Fimbrium – **decrease*****  CA1- **decrease*****  DG – **decrease***** | **Low dose**:  Fimbrium – **increase***** CA1 – **increase***** DG – **increase***** | Direct effect of FTY720 on S1PR5 not detectable at low dose, but detectable at high dose. EAE decreases S1PR5 expression levels, but FTY720 upregulates S1PR5 in the presence of EAE from low dose. |
|  | **High dose**:  Fimbrium – **increase*****  CA1 – **increase***** DG – **increase***** |  | **High dose**:  Fimbrium – **increase*****  CA1 – **increase***** DG – **increase***** |  |
